# Natural Phosphodiesterase-4 Inhibitors with Potential *Anti*-Inflammatory Activities from *Millettia dielsiana*

**DOI:** 10.3390/molecules28217253

**Published:** 2023-10-25

**Authors:** Vu Thi Thu Le, Hoang Van Hung, Nguyen Xuan Ha, Cao Hong Le, Pham Thi Hong Minh, Do Tien Lam

**Affiliations:** 1 Thai Nguyen University of Agriculture and Forestry, Quyet Thang, Thai Nguyen 24119, Vietnam; vuthithule@tuaf.edu.vn (V.T.T.L.);; 2 Thai Nguyen University-Lao Cai Campus, Thai Nguyen University, Lao Cai City 31000, Vietnam; 3 Institute of Natural Products Chemistry, Vietnam Academy of Science and Technology, 18 Hoang Quoc Viet, Cau Giay, Hanoi 10072, Vietnam; 4 Faculty of Chemistry, Graduate University of Science and Technology, Vietnam Academy of Science and Technology, 18 Hoang Quoc Viet, Cau Giay, Hanoi 10072, Vietnam

**Keywords:** *Millettia dielsiana*, phosphodiesterase-4 inhibitors, *anti*-inflammatory, MD modeling

## Abstract

The results of in silico screening of the 50 isolated compounds from *Millettia dielsiana* against the target proteins PDE4 (PDE4A, PDE4B, and PDE4D) showed binding affinity ranges from −5.81 to −11.56, −5.27 to −13.01, and −5.80 to −12.12 kcal mol^−1^, respectively, with median values of −8.83, −8.84, and −8.645 kcal mol^−1^, respectively. Among these compounds, Millesianin F was identified as the most promising PDE4A inhibitor due to its strongest binding affinity with the target protein PDE4A. (−11.56 kcal mol^−1^). This was followed by the compound 5,7,4′-trihydroxyisoflavone 7-*O-β*-d-apiofuranosyl-(1→6)-β-d-glucopyranoside (D50) with the binding affinity value of −11.35 kcal mol^−1^. For the target protein PDE4B, compound D50 exhibited the strongest binding affinity value of −13.01 kcal mol^−1^, while showing poorer inhibition ability for PDE4D. The 100 ns MD simulation examination (radius of gyration, Solvent Accessible Surface Area (SASA), Root-Mean-Square Deviation (RMSD), Root-Mean-Square Fluctuation (RMSF), and hydrogen bonding) was carried out to examine the overall stability and binding efficiency of the protein–ligand complex between compounds (Millesianin F, Millesianin G, Claclrastin-7-*O-β*-d-glucopyranoside, 7-hydroxy-4′,6 dimethoxyisoflavone-7-*O-β*-d-apiofuranosyl-(1→6)-*β*-d-glucopyranoside, 7-hydroxy-4′,8-dimethoxyisoflavone 7-*O-β*-d-apiofuranosyl-(1→6)-β-d-glucopyranoside, Odoratin-7-*O-β*-d-glucopyranoside, and 5,7,4′-trihydroxyisoflavone 7-*O-β*-d-apiofuranosyl-(1→6)-*β*-d-glucopyranoside) and PDE4 (A, B) subtype proteins. Compound D50 has shown strong *anti*-inflammatory activity, as evidenced by experimental results. It effectively inhibits PDE4B and PDE4D, with IC_50_ values of 6.56 ± 0.7 µM and 11.74 ± 1.3 µM, respectively. Additionally, it reduces NO production, with an IC_50_ value of 5.40 ± 0.9 µM. Based on these findings, it is promising and considered a potential novel *anti*-inflammatory drug for future development.

## 1. Introduction

Phosphodiesterase-4 (PDE4) plays a special role in the control of cell function, critical regulators of intracellular 3′,5′-cyclic adenosine monophosphate (cAMP) levels, cAMP signaling, and signal compartmentalization. By increasing cAMP levels, PDE4 inhibitors show a broad spectrum of *anti*-inflammatory effects in almost all inflammatory cells. A vast array of PDE4 inhibitors have been evaluated in clinical trials for various inflammatory conditions. Many drugs have considerable efficacy but also have adverse effects such as nausea and emesis, which limit their dosing and subsequently their immunomodulatory activity. Thus, developing PDE4 inhibitors that retain *anti*-inflammatory effects while limiting side effects has been a significant focus of pharmaceutical research for treating inflammatory diseases [1,2].

PDE4 is the major family of PDE enzymes expressed in immune and inflammatory cells. Inhibition of PDE4 has been shown to suppress a diverse spectrum of inflammatory responses in vitro and in vivo. More importantly, many PDE4 inhibitors in development are efficacious in animal models of various inflammatory disorders, such as asthma, COPD, psoriasis, inflammatory bowel diseases, rheumatoid arthritis and in clinical trials for asthma and COPD. These data thus provide strong evidence that PDE4 is a valid, promising drug target for different inflammatory conditions [1,2].

*Millettia* plants have a reputation for being valuable therapeutic herbs and are frequently utilized in folk medicine. There are 260 species in the genus *Millettia*, a member of the Fabaceae family, found throughout the world’s tropical and subtropical regions. Of these, *Millettia* species are distributed in Africa, Asia, and Vietnam with 130, 121, and 20 identified species, respectively [3,4]. Otherwise, plants belonging to this genus have been used in folkloric medicine to treat various ailments, including wound healing, back pain, blood circulation issues, skin diseases, rheumatic arthritis, and gynecological diseases [5,6]. Additionally, various chemical constituents in the genus *Millettia* are members of the flavonoid, including isoflavones (approximately 100 compounds), flavones (about 30 compounds), flavanones (about 20 compounds), and some other chemical groups, including flavanonol, rotenoid, and chalcone (approximately 30 compounds) [7]. Numerous biological effects of flavonoids, including *anti*-inflammatory properties, vascular stability, *anti*-toxicity, protection of liver function, antioxidant capabilities, antiviral activity, and potential cancer-fighting properties [8,9], have been demonstrated.

*Millettia dielsiana* Harms ex Diels belonging to the genus *Millettia* and the Fabaceae family is an important medicinal herb, especially in traditional medicine, and plays an important role in modern medicine. To date, about 50 compounds were isolated and identified from *Millettia dielsiana* and most of these isolated compounds belong to flavonoids. Haoyu Ye et al. conducted a study to evaluate the *anti*-inflammatory activity of eleven isoflavones. The results showed that compound millesianin C strongly inhibited NO production with IC_50_ value of 1.37 μM, compounds durallone, barbigerone, ichthynone, durmillone, methoxicalpogonium isoflavone A, calopogonium isoflavone A, millesianin D, and millesianin I exhibited moderate inhibition with IC_50_ values in the range of 1.63–2.22 μM [10]. The results of the evaluation of the ability to inhibit NO production in RAW264.7 cells induced by LPS, using 15 isolated compounds from the stem of *Millettia dielsiana*, showed that the compound (3S)-vestitol exhibited strong inhibitory activity with an IC_50_ value of 16.0 μM. Next, isoliquiritigenin and tupichinol C exhibited moderate activity against NO production with IC_50_ values of 31.2 and 38.4 μM, respectively [11]. These previous reports evidenced that *Millettia dielsiana* contains active compounds with potential *anti*-inflammatory effects.

This study contributes to discovering active phosphodiesterase-4 inhibitors for treating inflammatory diseases. We conducted an in silico study on 50 compounds from *Millettia dielsiana*, which was further validated through in vitro experiments.

## 2. Results and Discussion

### 2.1. In Silico Molecular Docking Simulations

A computer-aided drug design method known as molecular docking aims to forecast possible interactions between a protein and one or several ligands [12]. This method determines the interaction mode and binding affinity represented by a scoring function, and the ideal spatial conformation and functional activity [13]. In this study, the molecular docking method was conducted to assess the action of the potent *anti*-inflammatory activity of 50 isolated compounds from *Millettia dielsiana* towards targeting PDE4 subtype proteins.

**Figure 1:**
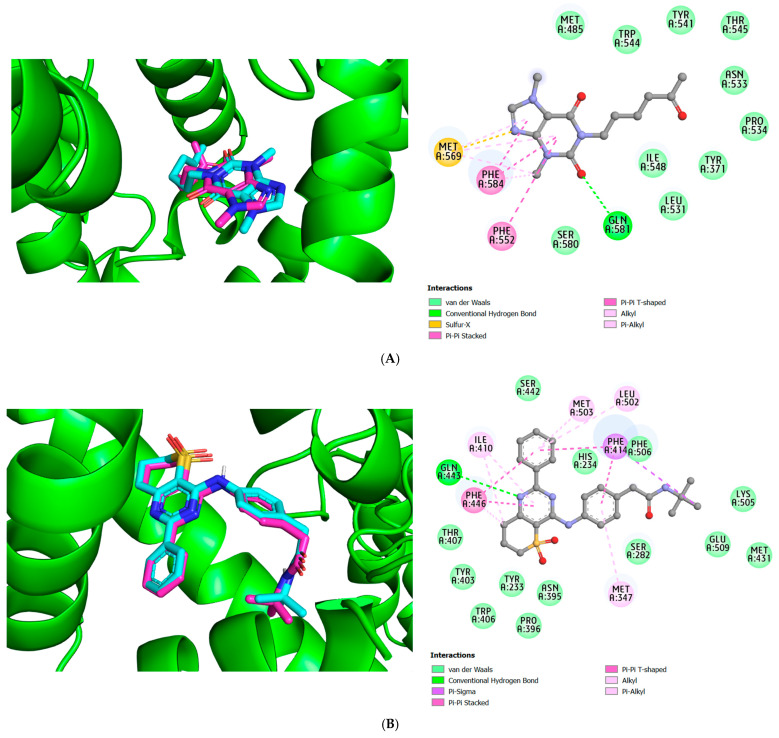

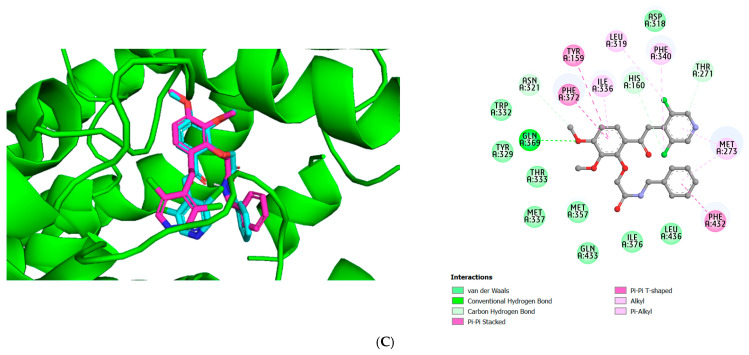
Re-docking of the co-crystallized ligands, pentoxifylline for PDE4A (**A**), NVW for PDE4B (**B**), and 3KQ for PDE4D proteins (**C**), was performed in their respective active-site pockets. The native ligands are shown in cyan, while the docked ligands are displayed in magenta.

Before beginning any virtual screening investigation, docking validation research should be performed. The purpose of docking validation was to confirm the reliability of the docking approach concerning conformational 3D ligands for the target protein. The root means square deviation (RMSD) was computed for this docking validation process. A measure called the RMSD value is used to compare two structures and determine how similar they are based on differences in atomic distance. The co-crystallized ligand and the lowest energy pose acquired upon re-docking were superimposed as in Figure 1. The RMSD must be within the reliable range of 2 Å for the docking operation to be validated. In this section, the RMSD value obtained to 0.977534 Å (PDE4A), 0.449372 Å (PDE4B), and 1.37339 Å (PDE4D), which is less than 2Å; therefore, the docking methodology utilizing AutoDock Vina is suitable for virtual screening research of the isolated compounds from *Millettia dielsiana* against the PDE4 target proteins. PDE4A, -B, and -D subtypes of PDE4 are predominantly found in immunological and inflammatory cells, including T cells, B cells, macrophages, monocytes, neutrophils, and eosinophils [14]. Therefore, these subtypes were selected for the current study.

**Figure 2:**
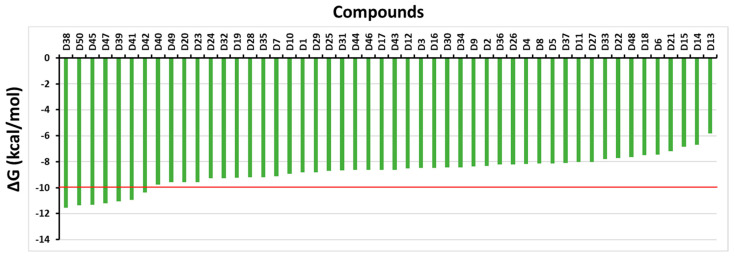
Distribution of the binding affinity between 50 compounds and the PDE4A protein. The outcomes were obtained with the Gnina program.

Figure 2 shows the affinity binding of the studied compounds docked into the active-site pocket of the PDE4A receptor. The estimated binding affinity for 50 compounds ranges from −5.81 to −11.56 kcal mol^−1^ and the median value of −8.83 kcal mol^−1^ (Table S2). The seven compounds that were chosen had significant binding affinities (the cutoff value as −10.00 kcal mol^−1^) for the PDE4A protein when compared to the reference compound pentoxifylline (−6.09 kcal mol^−1^). However, a thorough investigation of the seven top-lead compounds’ potential docked conformations was conducted to examine their interactions with the PDE4A-binding pocket is detailed in Table 1, and Figure S1 illustrates the precise relationship between PDE4A and the entire set of top-lead compounds. Note that the compounds D38, D47, D41, D39, and D42 were observed to form a hydrogen bond with the crucial amino acid GLN581, establishing a similar interaction as described for pentoxifylline in Figure S1. The bond length between D41 and GLN581 residue is the shortest with a distance of 2.02 Å and it is also strongest hydrogen bonding that was formed from the binding pose of D41 and active pocket on the PDE4A protein, as demonstrated in Table 1.

Furthermore, compound D38 showed the strongest binding affinity with the PDE4A protein target (−11.56 kcal mol^−1^) as shown in Table 1. Considering the found binding mode, compound D38 docked into PDE4A’s binding cavity and interacted with the same binding sites with the co-crystallized ligand pentoxifylline. Notably, conventional hydrogen bond interactions between the compound D38 and residues such as GLU442, ASP413, HIS412, ASP530, TYR371, ASN533, and GLN581. The π-electrons of the rings interacted with PHE584 through π-π stacked interactions. Furthermore, compound D38 also engages in π-alkyl interactions with MET485 and MET569. This compound was thought to be a good enzyme inhibitor using the ligand interaction model for both thermodynamic and interaction sites. As a result, D38 interacted well with the active enzyme pocket of PDE4A.

**Figure 3:**
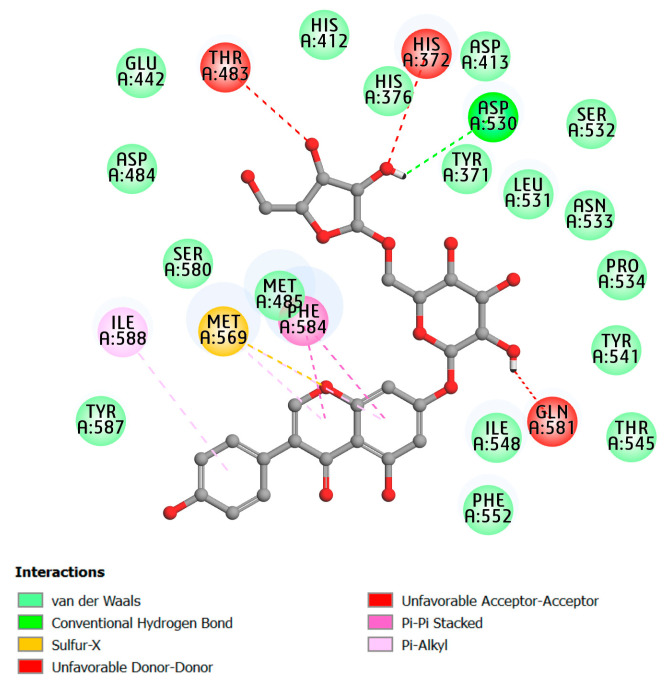
Two-dimensional representation of binding interaction of the compound D50 and the amino acid residues of PDE4A enzyme (PDB ID: 3TVX).

Docked pose for the conformation of the ligand in the D50 compound anchored to PDE4A protein with the binding affinity values of −11.35 kcal mol^−1^, as shown in Table 1. This pose formed a hydrogen bond with the ASP530 amino acid residue at a distance of 2.68 Å. An aromatic ring of pose was found to be the capping unit through π-alkyl (hydrophobic contact) from ILE588 to ring B of an isoflavone skeleton. In addition, D50 interacts via a sulfur–X bond with the MET569 amino acid residue and forms a π–π stack with the PHE584 amino acid residue, as illustrated in Figure 3.

The obtained results suggest that the remaining top leads D45, D47, D41, D39, and D42 identified by the docking method bind to the substrate-binding cleft of PDE4A protein via various hydrogen bonds and hydrophobic interactions. These substances form a variety of sidechain hydrogen bonds with several crucial residues, including GLN581, ASP530, ASP413, HIS416, VAL419, TYR371, and ASN533 of the PDE4A protein. It should be noted that the ASN395-ILE450 region is essential and more favorable for inhibitors to bind to PDE4 [15]. Also, it has been established that the hydrophobic and hydrogen-bonding interactions between PDE4 and inhibitors occur in the region Leu400-Ile450 [16].

**Figure 4:**
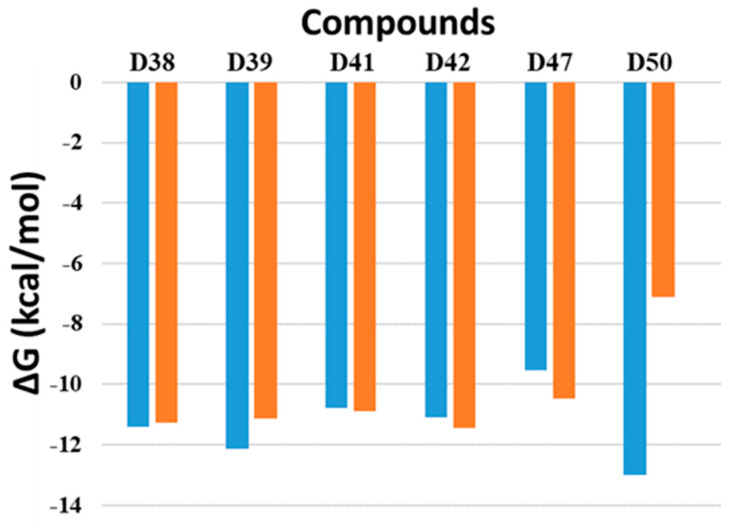
Binding affinities between selected compounds and PDE4 isoforms (PDE4B (orange) and PDE4D (blue)).

**Figure 5:**
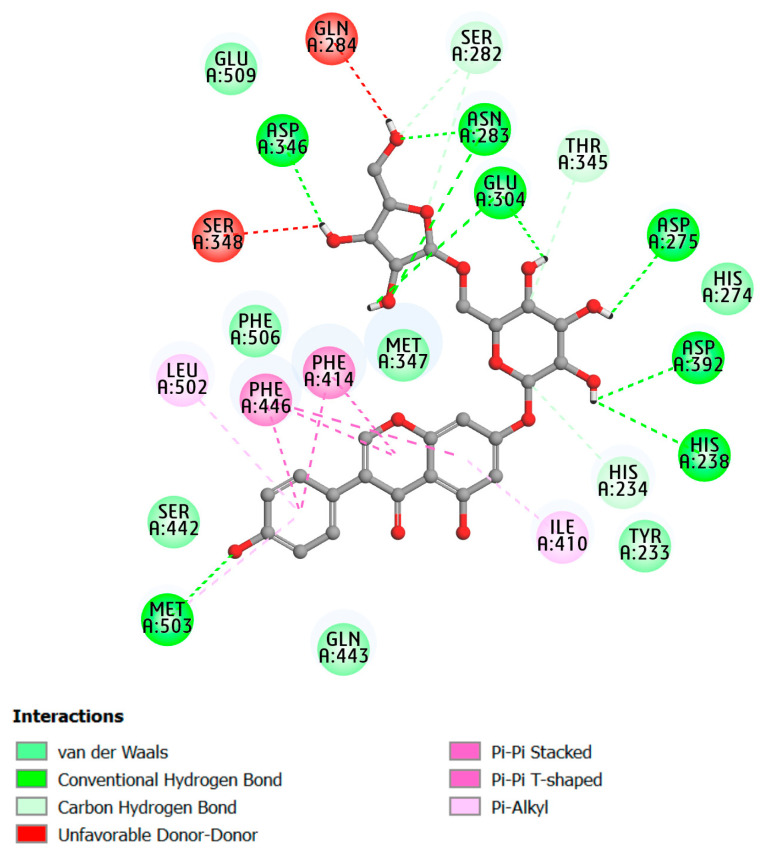
Two-dimensional representation of binding interaction of the compound D50 and the amino acid residues of PDE4B enzyme (PDB ID: 3W5E).

PDE4D is found in the area postrema and nucleus of the solitary tract, whereas PDE4B is expressed in immunological, inflammatory, and airway smooth muscle cells. Because PDE4D inhibition causes the emetic reaction of PDE4D inhibitors, PDE4B inhibitors are anticipated to be a promising therapeutic option for the treatment of inflammatory diseases [17,18,19]. Therefore, in this study, the selectivity of PDE4B was assessed through molecular docking analysis at the active site of both PDE4B and PDE4D. The results are presented in Figure 4, Table 2, Tables S2 and S3. The binding affinities estimated for PDE4B ranged from −5.27 to −13.01 kcal mol^−1^, whereas for PDE4D, they ranged from −5.8 to −12.12 kcal mol^−1^. Six top-lead compounds, namely D38, D39, D41, D42, D47, and D50, exhibited binding strengths potential against PDE4B protein, as indicated by the binding affinity values below the threshold of −10.00 kcal mol^−1^. This observation is consistent with studies on PDE4A inhibition and justifies the selection of these compounds for evaluating PDE4B selectivity. As shown in Figure 4, most of the inhibitory compounds displayed similar binding affinities for both receptors. However, compound D50 exhibited the highest binding affinity for PDE4B compared to other compounds and reference compounds, while demonstrating weaker binding strength against PDE4D, indicating a higher selectivity towards PDE4B over PDE4D. Therefore, the fundamental binding interactions of this compound with the PDE4B receptor were further analyzed to explore the structural features contributing to the selectivity of PDE4B. Table 2 displays the docked pose for the conformation ligand of the D50 compound, which is attached to PDE4B protein and has a binding affinity of −13.01 kcal mol^−1^. Seven hydrogen bonds were made by this pose with the amino acid residues ASP346, ASN283, GLU304, ASP275, ASP392, HIS238, and MET503 at distances of 2.12, 3.18 (3.36), 2.67, 2.46, 2.45, 2.72, and 3.15 Å, respectively. Additionally, hydrophobic interactions such as pi–pi T-shaped, stacked, and pi–alkyl interactions were observed at positions PHE414, PHE446, LEU410, and LEU502 (Figure 5). The details of the interactions including the top six compounds are further elucidated and presented in Table 2.

### 2.2. Analysis of Molecular Dynamics

**Figure 6:**
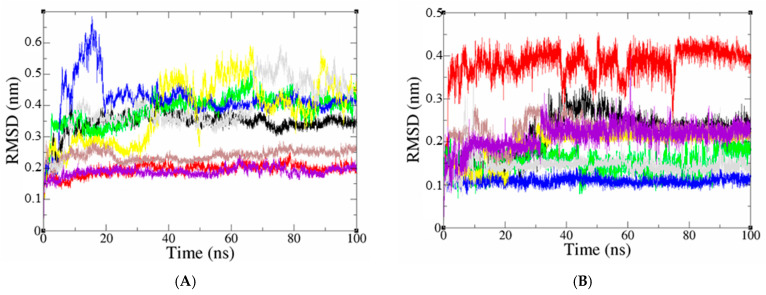

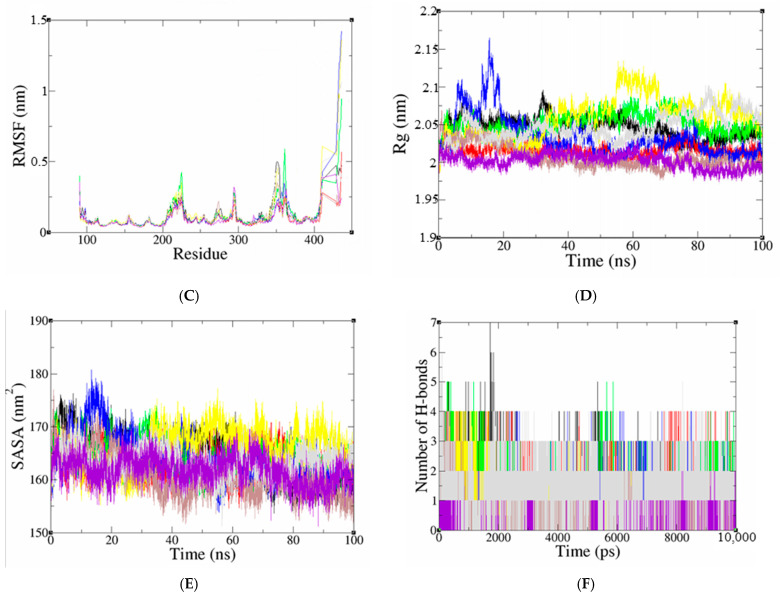
(**A**) The plot of RMSD values for the protein backbone; (**B**) The plot of RMSD values for the docked ligand; (**C**) the root mean square fluctuation (RMSF); (**D**) The radius of gyration (Rg) of a protein in the presence of ligands and reference compound; (**E**) Inter H-bond formation between PDE4A protein and ligands (**F**) the number of H bonds formed between the ligand and the crystal structure of protein determined using 100 ns simulation trajectories. The compounds D38, D39, D41, D42, D45, D47, D50, and pentoxifylline are black, red, green, blue, yellow, brown, grey, and violet, respectively.

The docked pose of top leads was selected to analyze the stability in the virtual physiological environment with PDE4 proteins. Using MD modeling, the ligand’s capacity to bind to the protein overtime at the atomic level was evaluated. The radius of gyration, SASA, RMSD, RMSF, and hydrogen bonding, all contribute to the understanding of the protein–ligand complex’s bond pattern and structural change. A 100 ns MD simulation examination of the conformational stability of the ligand with the 3D structure of the receptor (PDB: 3TVX, 3W5E, and 4WCU) was carried out to examine the overall stability and binding efficiency of the protein–ligand complex. The conformational stability of a system is determined via RMSD parametric analysis, which provides detailed structural information. As shown in Figure 6A, the calculated RMSD values for the PDE4A protein, with reference to its backbone (RMSD backbone), showed an overall typical behavior for MD simulations. Due to removing constraints at the start of the MD simulation run, the RMSD backbone trajectories increase over the initial frames. Compound D39 stabilized after the first time of MD simulation, with steady trajectories showing RMSD values leveling off at roughly 0.19 nm until the end of the simulation course. Furthermore, the RMSD result of the D39-binding protein was similar to that of the pentoxifylline-binding protein in terms of structural stability. The conformation changes during 25 ns and 20 ns of compounds D47 and D42, respectively, and then reaches equilibrium and steady-state with mean RMSD values of 0.24 nm and 0.42 nm, respectively. In addition, the protein binding compounds D45, D50, and D41 have significant structural changes indicating their instability. The RMSD of the docked ligand in the complex indicated that the ligand was stable throughout the MD simulations of the protein–ligand complex. The RMSD value for the compounds D41 and D42 to dock in the active sites of PDE4A protein was stable during the simulations compared to the reference compound. After the 13 ns simulation, compound D50 presented straight lines with reduced deviation levels. Meanwhile, the compounds D38, D45, and D47 have structural changes that are almost similar to those of the pentoxifylline compounds. Compound D39 exhibits mostly steady fluctuations, with an average RMSD value of 0.38 nm but with minor changes over a 40–60 ns time scale.

**Figure 7:**
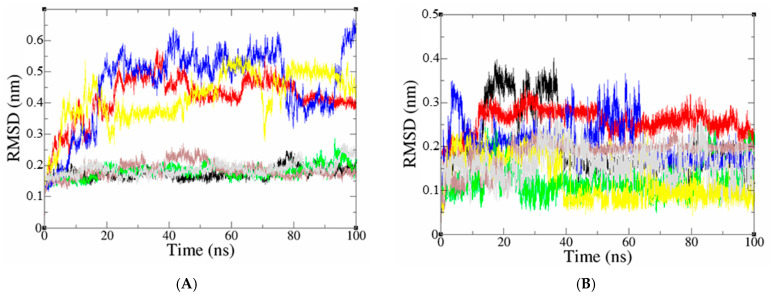

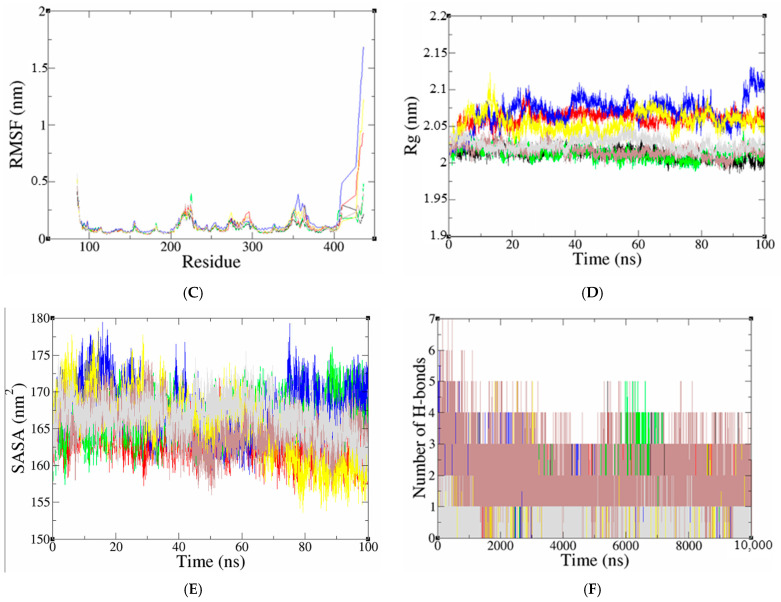
(**A**) The plot of RMSD values for the protein backbone; (**B**) The plot of RMSD values for the docked ligand; (**C**) The root mean square fluctuation (RMSF); (**D**) The radius of gyration (Rg) of a protein in the presence of ligands and reference compound; (**E**) Inter H-bond formation between PDE4B protein and ligands; (**F**) The number of H bonds formed between the ligand and the crystal structure of protein determined using 100 ns simulation trajectories. The compounds D38, D39, D41, D42, D45, D47, D50, and NVW are black, red, green, blue, yellow, brown, grey, and violet, respectively.

In the molecular dynamics research of PDE4B complexes, focusing on the six most promising compounds, as depicted in Figure 7A, the average RMSD backbone values for the complexes D38, D39, D41, D42, D47, D50, and NVW were found to be 0.18 nm, 0.42 nm, 0.18 nm, 0.46 nm, 0.42 nm, 0.19 nm, and 0.19 nm, respectively. According to the RMSD backbone plot, the protein structure in complexes D38, D41, and D50 remained stable throughout the simulation, exhibiting only minor fluctuations within a range of 0.05 nm. These results are consistent with the reference inhibitor compound NVW. Conversely, the protein structure in complexes D39, D42, and D47 displayed significant variations in RMSD backbone values, indicating their instability during the 100 ns MD simulation period. The analysis of the RMSD graph for ligands in Figure 7B indicates that compound D50, which binds to the active site of the PDE4B protein, exhibits a significantly higher level of stability during the simulation compared to the reference compound. In contrast, D38 and D42 display substantial fluctuations, but eventually reach a stable state at approximately 40 ns and 70 ns, respectively, after the initiation of the simulation. These compounds maintain their stability throughout the remaining simulation duration. The RMSD values of ligand D47 illustrate an initial increase up to 0.25 nm, followed by a gradual decrease to 0.1 nm during the simulation. On the other hand, compound D41 exhibits structural variations that compromise its stability towards the end of the simulation. Ligand D39 demonstrates minor fluctuations, with an RMSD increase of approximately 0.1 nm observed from the 10 ns mark throughout the entire simulation period.

Root means square fluctuation (RMSF), which is depicted in Figure 6C and Figure 7C, measures the flexibility of protein amino acid residues in the presence of a ligand. The protein–ligand complex fluctuation pattern is remarkably similar, supporting the simulation’s findings that mobility was constrained for 100 ns. On the other hand, Figure 6C and Figure 7C show that the docked complexes’ maximal amino acid residues had RMSF values that were less than 1.5 nm, indicating a lesser level of flexibility.

The radius of gyration (Rg) of compounds was computed, which compares the shape of the protein at each time point to the hydrodynamics radius that can be obtained experimentally. The mean Rg values of protein for PDE4A-D38, PDE4A-D39, PDE4A-D41, PDE4A-D42, PDE4A-D45, PDE4A-D47, PDE4A-D50, PDE4A-reference were 2.05, 2.02, 2.05, 2.03, 2.06, 2.00, 2.05, and 2.00 nm, respectively, as shown Figure 6D. The Rg values of PDE4A binding with D39 and D47 as well as reference ligands (ranging from 1.98 to 2.05 nm) indicate low fluctuations and high stability in the system. In addition, during the first 20 ns of the simulation, Rg values of the D42 compound were observed with significant fluctuations. However, after this time, the complex PDE4A-D42 indicated different behaviors, with a reduction in the Rg value and stably folded after the simulation, which remained at a constant value during the 100 ns test period. The compounds D38 and D41 exhibited stability during the simulation. For PDE4B protein complexed with compounds D38, D39, D41, D42, D47, D50, and the reference compound NVW, the average Rg values of the protein are 2.01, 2.06, 2.01, 2.07, 2.06, 2.06, 2.02, and 2.03 nm, respectively. As shown in Figure 7D, small fluctuations within a range of 0.15 nm in Rg during the MD simulation time indicate a slight opening and closing of the N and C-terminal regions of the protein. Moreover, compounds D38, D41, and D50 exhibit high stability throughout the 100 ns simulation period, and this result is also consistent with the reference compound NVW.

The surface-accessible solvent area (SASA) variable is calculated over the entire trajectory for all systems to evaluate the protein surface area that is accessible to the solvent in which it is simulated. The surface area is defined as expanding when the SASA value is higher and as contracting when the SASA value is lower. The total SASA values for PDE4A-D38, PDE4A-D39, PDE4A-D41, PDE4A-D42, PDE4A-D45, PDE4A-D47, and PDE4A-D50 complexes were 164.3291, 163.0158, 164.0624, 164.2352, 166.0155, 160.6065, and 163.2928 nm^2^, respectively. Meanwhile, PDE4B-D38, PDE4B-D39, PDE4B-D41, PDE4B-D42, PDE4B-D45, PDE4B-D47, and PDE4B-D50 complexes were 163.624, 164.6477, 166.799, 167.8632, 165.4084, 165.2088, and 166.7438 nm^2^, respectively. Both the protein docked with the appropriate reference inhibitors and the compounds being studied in this study had SASA values that were noticeably similar. The profiles of the docked complexes were good since they did not vary significantly.

The primary marker of specificity and molecular interactions between the protein and inhibitor complexes is hydrogen bond formation. The hydrogen bonds (H-bonds) formed for PDE4 subtype proteins with respective studied compounds were observed for entire trajectories using 100 ns MD simulation trajectories as depicted in Figure 6F and Figure 7F. The number of hydrogen bonds observed between a protein and ligand complex ranges from 0 to 7 H-bonds. Hence, the ligands’ binding poses to the PDE4 (A, B) isoforms were relaxed to stable states. The amino acid residues ASP530, ASN533, TYR371, GLN581, GLU442, THR483, and Gln210 for PDE4A protein and ASN283, ASP275, GLU304, ASP392, GLN443, HIS238, and GLU509 for PDE4B protein were found after refined 100 ns MD trajectories (Figure 7F, Figures S3 and S4) that crucial residues regulating the binding process. In addition, it was discovered that all studied compounds were able to form rigid non-bonded contacts—hydrogen bonds and hydrophobic contacts—to PDE4 (A, B) isoforms more effectively than the reference compounds. It could help to explain why these substances adopt higher binding affinities than the reference compounds. Furthermore, these results contributed to the stability of the protein–ligand combination by maintaining a stable state throughout the simulation.

### 2.3. Toxicity Analysis

Based on our knowledge, there is currently no report on the toxicity determination of the chemical constituents of *Millettia dielsiana*; as such, the information toxicity definition is almost nonexistent. Thus, in silico prediction methods were used in this study to predict the toxicity of compounds found in *Millettia dielsiana*, using the ProTox-II web server. It should be noted that, according to the obtained predictions, all the selected compounds showed a predicted LD_50_ value of 5000 mg/kg, and their toxicity class was designated as 5. Therefore, these compounds of *Millettia dielsiana* have a toxicity level that is almost safe for oral administration, compared to reference compounds (Table S4), NVW, 3KQ, and pentoxifylline being more toxic with a toxicity level of 4 and lower LD_50_ concentrations (LD_50_ = 2000 mg/kg and LD_50_ = 780 mg/kg, respectively). Toxicity predictions for organ toxicity (hepatotoxicity) were assessed for the different compounds determined, as the liver is the organ where these toxins are metabolized. The results have been presented in Table S5. It is worth noting that compound D50 has no toxicity against any of the studied targets (all inactive).

Regarding organ toxicity, the results obtained show that all compounds were predicted not to be toxic to the liver (hepatotoxicity). Furthermore, concerning different toxicity endpoints studied, compounds D38, D39, D41, D42, D45, and D47, were observed to be immunotoxic. Except for this toxicity, compounds D39, D42, and D45 did not cause any additional toxicity on other targets, while compound D41 was predicted to be a cytotoxic compound.

In addition, the obtained prediction pathways for toxicological, stress response, and nuclear receptor signaling were reported accordingly [20]. Computational estimates showed that compound D41 might interact with the aryl hydrocarbon receptor (AhR) in the nuclear receptor signaling pathways. Compound D38 demonstrated activity in interacting with the target phosphoprotein p53 (tumor suppressor) in the regarding stress response pathways.

### 2.4. Evaluation of In Vitro Anti-Inflammatory Compounds (D15 and D50)

**Figure 8:**
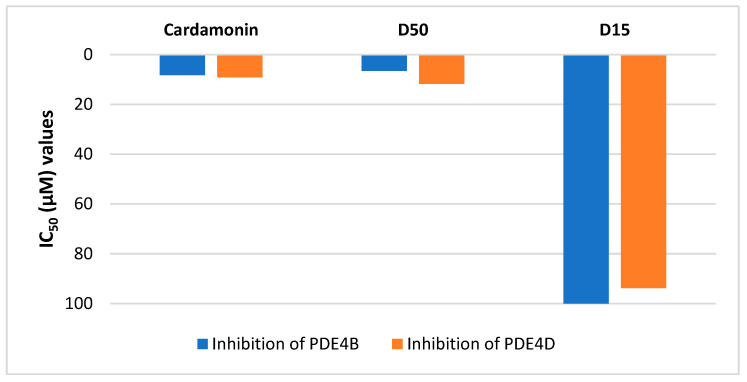
The IC_50_ (µM) values of inhibition of PDE4B/D of compounds D15 and D50. Data are of an average of three determinations ± SEM. Control (+) is a Cardamonin. The compounds 5,7,4′-trihydroxyisoflavone 7-*O*-*β*-d-apiofuranosyl-(1→6)-*β*-d-glucopyranoside and *trans*-3-*O*-*p*-hydroxycinnamoyl ursolic acid were named as D50 and D15, respectively.

**Figure 9:**
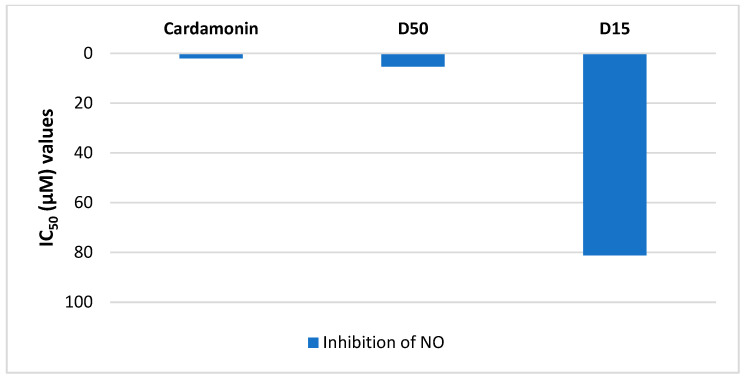
The IC_50_ (µM) values of inhibition of NO production of compounds D15 and D50. The highest concentration of the compounds was 50 µM for NO inhibition on RAW264.7 cells. Data are of an average of three determinations ± SEM. Control (-) is a DMSO and control (+) is a Cardamonin. The compounds 5,7,4′-trihydroxyisoflavone 7-*O*-*β*-d-apiofuranosyl-(1→6)-*β*-d-glucopyranoside and *trans*-3-*O*-*p*-hydroxycinnamoyl ursolic acid were named as D50 and D15, respectively.

The in vitro *anti*-inflammatory activity of compounds (D15 and D50) obtained from the stems of *Millettia dielsiana* were evaluated through the inhibition of PDE4B and PDE4D. The experimental data were recorded and shown in Figure 8. Furthermore, it is possible to compare the experimental results (IC_50_ values) correlation of in vitro *anti*-inflammatory activity between the methods of inhibition of PDE4B/D, and NO production (Figure 8 and Figure 9).

The 5,7,4′-trihydroxyisoflavone 7-*O*-*β*-d-apiofuranosyl-(1→6)-*β*-d-glucopyranoside (D50) showed a potent PDE4B and PDE4D inhibitors with an IC_50_ of 6.56 ± 0.7 and 11.74 ± 1.3 μM, respectively; and *trans*-3-*O*-*p*-hydroxycinnamoyl ursolic acid (D15) did not show activity (Figure 8). The compound 5,7,4′-trihydroxyisoflavone 7-*O*-*β*-d-apiofuranosyl-(1→6)-*β*-d-glucopyranoside (D50) exhibited the strongest in vitro inhibitory activity on NO production on RAW264.7 macrophages with the IC_50_ value of 5.40 µM and *trans*-3-*O*-*p*-hydroxycinnamoyl ursolic acid (D15) exhibited moderate *anti*-inflammatory activity with IC_50_ value 81.23 µM (Figure 9). The experimental IC_50_ values of compounds (D15 and D50) between the methods of inhibition of PDE4B, PDE4D, and NO production are similar.

Overall, the compound 5,7,4′-trihydroxyisoflavone 7-*O*-*β*-d-apiofuranosyl-(1→6)-*β*-d-glucopyranoside (D50) demonstrated much stronger bioactivities (*anti*-NO activity, inhibitory effect against PDE4B and PDE4D) than those of *trans*-3-*O*-*p*-hydroxycinnamoyl ursolic acid (D15). In addition, this 5,7,4′-trihydroxyisoflavone 7-*O*-*β*-d-apiofuranosyl-(1→6)-*β*-d-glucopyranoside (D50) showed comparable bioactivities to those of Cardamonin. Currently, the compound D50 was purified from *Millettia dielsiana* extract and found as potential antioxidant and anticancer effects via in vitro tests, and this compound was also confirmed as a promising inhibitor against PI3K/mTOR [21]. However, the potential *anti*-inflammatory effects of this constituent were new records of this study.

The difference in experimental IC_50_ values of compounds (D15 and D50) above is similar to the difference in virtual screening affinity binding values (Figure 2, Table 1, Table 2 and Tables S1–S3). This has contributed to confirming the agreement of results between experimental and virtual screening. However, more in-depth studies are needed.

## 3. Materials and Methods

### 3.1. Materials

Two compounds: 5,7,4′-trihydroxyisoflavone 7-*O*-*β*-d-apiofuranosyl-(1→6)-*β*-d-glucopyranoside (D50) and *trans*-3-*O*-*p*-hydroxycinnamoyl ursolic acid (D15) were purified and identified from *Millettia dielsiana* extract in the previous report [21].

### 3.2. Docking Studies

The molecular docking with deep learning studies was performed utilizing the GNINA v1.0.2 program [22] with a scoring function based on AutoDock Vina [23] to explore the binding mode of the isolated compounds toward PDE4 [24]. The 3D crystal structures of the target protein PDE4A, PDE4B, and PDE4D were downloaded from the RCSB protein data bank (https://www.rcsb.org/ accessed on 23 March 2023), with PDB IDs: 3TVX, 3W5E, and 4WCU, and resolutions: 2.84 Å, 2.30 Å, and 2.35 Å, respectively. The protein preparation steps included the removal of water molecules, co-factors, and metal ions and the addition of partial Kollman charges after associating hydrogen atoms by AutodockTools v1.5.6 software (http://mgltools.scripps.edu/, accessed on 23 March 2023). The chemical structures of 50 isolated compounds from *Millettia dielsiana* Harms were collected from previously published literature [11,25,26,27,28]. Those structures were drawn using ChemSketch 2021.1.2 software [ACD/ChemSketch, version 2021.1.2, Advanced Chemistry Development, Inc., Toronto, ON, Canada, www.acdlabs.com, 2021] and saved as CDX format files. The 2D sketched compounds were converted to 3D structures by 3DView 2021.1.2 software, followed by energy minimization then prepared for docking. For parameter preparation, the grid box was centered on the active site pocket and set to an exhaustiveness of 400. As a result of these processes, the predicted docking pose for each ligand was visualized by Discovery Studio Visualizer v2021 software [BIOVIA, Dassault Systèmes, Discovery Studio Visualizer, San Diego, 2021] [29,30,31].

### 3.3. Molecular Dynamics Simulation

The top-docked poses with better binding affinity than the control compound were used in a molecular dynamics (MD) simulation employing the GROMACS v2021 software package [32,33] with an Amber99sb-ildn force field. As in the previous protocols [21], the MD simulation ran for 100 ns, starting with the PDE4 subtype proteins (PDE4A, PDE4B, and PDE4D) and the compounds in their anticipated docking poses. In the Periodic Boundary Conditions (PBC), the system was solvated with TIP-3P water molecules with a gap of 1.0 nm. Na^+^ and Cl^-^ were introduced into the system at constant pressure (1.0 bar) and temperature (300 K) to balance the overall charge. The coordinates of the ligands were prepared to transform them into topologies appropriate for GROMACS using AmberTools22 [34] and ACPYPE [35]. The B3LYP/6-31G(d,p) level of theory was used for quantum chemical calculations with an implicit solvent (ε = 78.4) to determine the chemical details of the selected compounds using the GAMESS package [36]. The restrained electrostatic potential (RESP) approach was employed to assign the atomic charges of the ligands [37]. For non-bonded interactions, the electrostatic interaction and the van der Waals (vdW) interaction were set as in our previous studies [21,38,39]. All bonds were restrained throughout simulations by the LINCS algorithm. The steepest descent method was first applied to minimize the protein–ligand complex system. Then, a minimized complex was performed to run with 100 ps of NVT and NPT ensemble. A harmonic force with a 1000 kJ mol^−1^ nm^−2^ spring constant restricted the complex’s atomic positions. The systems were finally relaxed for production MD. The root mean square deviation (RMSD), the radius of gyration (Rg), root mean square fluctuation (RMSF), Solvent Accessible Surface Area (SASA) were analyzed and plotted using Xmgrace v5.1.25 software.

### 3.4. Toxicity Prediction

Toxicity screening of selected compounds assists in stating their toxicity class, predicted LD_50_, organ toxicity (hepatotoxicity target), toxicological endpoints (immunotoxicity, mutagenicity, and cytotoxicity targets), toxicological pathways (Aryl hydrocarbon Receptor, Androgen Receptor, Androgen Receptor Ligand Binding Domain, Aromatase, Estrogen Receptor Alpha, Estrogen Receptor Ligand Binding Domain, and Peroxisome Proliferator-Activated Receptor Gamma targets). Selected phytochemicals in SMILES format from PubChem were loaded on the ProTox II web server [40] (https://tox-new.charite.de/protox_II/index.php?site=home, accessed on 23 March 2023). This web server combines machine learning and fragment cross-validation based on fragment similarity, most frequent characteristics, and chemical similarity. In addition, toxicity classes are outlined by the globally standardized method of classification in chemical labeling with six classes as follows: class I (fatal if swallowed with LD_50_ ≤ 5 mg/kg), class II (fatal if swallowed with 5 mg/kg < LD_50_ ≤ 50 mg/kg), class III (toxic if swallowed with 50 mg/kg < LD_50_ ≤ 300 mg/kg), class IV (harmful if swallowed with 300 mg/kg < LD_50_ ≤ 2000 mg/kg), class V (may be harmful if swallowed with 2000 mg/kg < LD_50_ ≤ 5000 mg/kg), and class VI (non-toxic with LD_50_ > 5000).

### 3.5. Anti-Inflammatory Activity Test Method

The *anti*-inflammatory activity in vitro of isolated compounds was carried out at the Institute of Natural Products Chemistry, Vietnam Academy of Science and Technology, Hanoi, Vietnam.

#### 3.5.1. In Vitro PDE4B/D Enzymatic Assay

Recombinant PDE4B/D was from Sigma Aldrich Co Ltd. A PDE4B/D inhibitors assay was performed using a cAMP dynamic 2 kit. Serial dilutions of isolated compounds with DMSO (100-fold of final concentration) were prepared. Compound solutions were diluted 20-fold with cAMP assay buffer (50 mM Tris, 6 mM MgCl_2_, pH 7.5). Enzyme solution (10 mL of 30 ng/mL or cAMP assay buffer for the control) and 5 mL of isolated compounds (5-fold concentration of final concentration) were added to each well of the 96-well micro assay plate and pre-incubated at room temperature for 10 min. The enzymatic reactions were started by adding 10 mL of cAMP solution (150 nM in cAMP assay buffer) or cAMP assay buffer and incubated at 37 °C for 30 min. The enzymatic reaction was terminated by placing the assay plate on ice. The concentration of cAMP was quantified by measurement of fluorescence intensity using Spectrophotometer Model-SP-2558 (Acton Research, Acton, MA, USA). The 50% inhibitory concentration (IC_50_) values were determined by nonlinear regression analysis and a sigmoidal dose–response equation using GraphPad Prism 4 (GraphPad Software Inc., La Jolla, CA, USA) [41,42].

#### 3.5.2. In Vitro *Anti*-Inflammatory Activity

Cell RAW264.7 (ATCC, Manassas, VA, USA) was cultured for 48 h in DMEM (Dulbecco’s Modified Eagle Medium) medium at 37 °C, 5% CO_2_, and 10% for fetal bovine serum (FBS). Then, the cell fluid was transferred to 96 wells with a density of 2.5 × 10^5^ cells/well. Cells were stimulated with 2 µL lipopolysaccharide (LPS 0.1 mg/mL) for 24 h and supplemented with different concentrations of drugs/reagents. Cardamonin is used as a control (+) and DMSO as a control (-). The suspension of the cell was incubated with Griess reagent, NaNO_2_ at different concentrations to build the calibration curve. Measure the reaction response at λ = 570 nm. The inhibition rate of NO (%) production is determined by the formula: %UC = ([X_TB_]_sample_ − [X_TB_]_LPS_)/([X_TB_]_ĐC_ − [X_TB_]_LPS_) × 100

In which [X_TB_] is the average NO concentration calculated based on the standard NaNO_2_.

The remaining cell portion after being used to evaluate in vitro activity was supplemented with MTT solution (0.5 mg/mL in PBS), incubated for 4 h in a 5% CO_2_ incubator at 37 °C. Formazan crystalline metabolites are dissolved in dimethyl sulfoxide (DMSO, Sigma-Aldrich, Saint Louis, MO, USA) and measured optical density at λ = 540/720 nm on Infinite F50 device (Tecan, Männedorf, Switzerland) [43,44].

## 4. Conclusions

Isoflavonoids, D38, 50, 45, 47, D41, D39, and D42 interacted well with the active enzyme pocket of PDE4 (PDE4A, PDE4B, and PDE4D). This has suggested good activity for the PDE4 enzyme in particular and *anti*-inflammatory activity in general. Further studies can focus on finding new *anti*-inflammatory agents from isoflavonoids. The chemical composition of *Millettia dielsiana*, which is rich in these active compounds, has contributed to clarifying the folk experience of treating arthritis and protecting the liver, and rheumatism.

All selected compounds from *Millettia dielsiana* showed a predicted LD_50_ of 5000 mg/kg and were almost safe by toxicity prediction in silico methods. Especially among them, compound D50 has no toxicity against any of the studied targets. Compound D41 was predicted to be cytotoxic, and might interact with AhR. And compound D38 demonstrated activity in interacting with p53. Determining the toxicity of compounds is necessary, an important step in drug design, and has great meaning in saving time, finances, and morality.

Compound 5,7,4′-trihydroxyisoflavone 7-*O*-*β*-d-apiofuranosyl-(1→6)-*β*-d-glucopyranoside (D50) may be suggested as a potent candidate for *anti*-inflammatory drug. This study primarily focuses on the in silico screening and in vitro experimental of *anti*-inflammatory activity. Therefore, research on in vivo activity, acute toxicity, and the correlation between them is a promising future research direction.

## Figures and Tables

**Table 1:** The docking scores of the top 7 compounds and their interactions with PDE4A protein.

Entry	Compounds Name	Affinity Binding (kcal mol^−1^)	Hydrogen Bonds	Distance of Hydrogen Bond (Å)	Hydrophobic Interaction
D38	Millesianin F	−11.56	GLU442 ASP413 HIS412 ASP530 TYR371 ASN533 GLN581	2.62 2.48 (2.26) 2.83 2.27 (3.02) 2.78 2.63 2.75	MET485, PHE584, MET569, GLN581
D50	5,7,4′-trihydroxyisoflavone 7-*O*-*β*-d-apiofuranosyl-(1→6)-*β*-d-glucopyranoside	−11.35	ASP530	2.68	ILE588, PHE584
D45	7-hydroxy-4′,8-dimethoxyisoflavone 7-*O*-*β*-d-apiofuranosyl-(1→6)-*β*-d-glucopyranoside	−11.32	GLN581 ASP530	2.74 2.52, 2.50	PHE552, PHE584, MET569, ILE588
D47	Odoratin-7-*O*-*β*-d-glucopyranoside	−11.19	GLN581	2.65	PHE584, MET569, MET485
D41	Claclrastin-7-*O*-*β*-d-glucopyranoside	−11.06	ASP530 ASP413 HIS416 VAL419 GLN581	4.75 2.62 2.18 2.61 1.85 (2.02)	PHE584, PHE552, ILE548, TYR371, HIS372
D39	Millesianin G	−10.94	ASP413 ASP530 TYR371 ASN533	2.37 (2.33) 2.93 (2.24) 2.81 2.68	MET485, PHE584, MET569, GLU442, HIS372
D42	7-hydroxy-4′,6 dimethoxyisoflavone-7-*O*-*β*-d-apiofuranosyl-(1→6)-*β*-d-glucopyranoside	−10.38	GLN581	2.62	MET485, PHE584, MET569

**Table 2:** The docking scores of the top 6 compounds and their interactions with PDE4B protein.

Entry	Compounds Name	Affinity Binding (kcal mol^−1^)	Hydrogen Bond	Distance of Hydrogen Bond (Å)	Hydrophobic Interaction
D50	5,7,4′-trihydroxyisoflavone 7-*O*-*β*-d-apiofuranosyl-(1→6)-*β*-d-glucopyranoside	−13.01	ASP346 ASN283 GLU304 ASP275 ASP392 HIS238 MET503	2.12 3.18 (3.36) 2.67 2.46 2.45 2.72 3.15	PHE432, MET273
D38	Millesianin F	−11.26	THR345 ASP392 TYR233 GLN443	2.38 2.61 2.14 (3.13) 3.30	PHE506, MET347, PHE414, ILE410, PHE446
D47	Odoratin-7-*O*-*β*-d-glucopyranoside	−10.86	ASP346 ASN283 GLU304 THR345 GLN443	2.96 3.20 2.48 2.42 2.71	MET347, PHE414, PHE446, PHE506, SER282
D41	Claclrastin-7-*O*-*β*-d-glucopyranoside	−10.77	TYR233 ASP392 HIS278 GLU304	2.35 2.60 2.65 2.94	PHE446, ILE410, PHE414, PHE506, TYR233
D39	Millesianin G	−10.73	ASP346 ASN283 HIS307 ASP275 HIS234	2.42 2.64 (3.34, 3.37) 2.64 3.02 3.00	MET503, PHE506, PHE446, PHE414, MET347
D42	7-hydroxy-4′,6 dimethoxyisoflavone-7-*O*-*β*-d-apiofuranosyl-(1→6)-*β*-d-glucopyranoside	−10.57	TYR233 ASP392 HIS238 ASP275 GLN443 HIS278 VAL281 ASN283	2.70 2.38 2.12 2.12 (2.67) 2.75 2.56 2.94 2.59	PHE414, ILE410, PHE446, MET347

## Data Availability

Not applicable.

## Supplementary Materials

The following supporting information can be downloaded at: https://www.mdpi.com/article/10.3390/molecules28217253/s1, Table S1: In silico Docking of isolated compounds from *Millettia dielsiana* against PDE4A protein. Table S2: In silico Docking of isolated compounds from *Millettia dielsiana* against PDE4B protein. Table S3: In silico Docking of isolated compoundsfrom *Millettia dielsiana* against PDE4D protein. Figure S1. 2D representation of binding interaction of the hit compounds and the amino acid residues of PDE4A enzyme (PDB ID: 3TVX). Figure S2. 2D representation of binding interaction of the hit compounds and the amino acid residues of PDE4B enzyme (PDB ID: 3W5E). Figure S3. 2D interaction diagrams of the studied compounds from MD-refined structures of these complexes with PDE4A were analyzed at a time of 100 ns. Figure S4. 2D interaction diagrams of the studied compounds from MD-refined structures of these complexes with PDE4B were analyzed at a time of 100 ns. Table S4. Acute oral toxicity prediction obtained by using the ProTox-II web server. Table S5. Organ toxicity, toxicological endpoints, and toxicological pathways predicted activity calculated using the ProTox-II web server.

## Sample Availability

Samples of compounds are available from the authors.
