# Supplementary material for: Natural Phosphodiesterase-4 Inhibitors with Potential Anti-Inflammatory Activities from Millettia dielsiana
Source: Molecules. 2023 Oct 25;28(21):7253. doi: 10.3390/molecules28217253 (PMC10650832; doi:10.3390/molecules28217253)
Supplement: Supplementary file 1 [file molecules-28-07253-s001.zip › molecules-2506595-supplementary.pdf]

Article

Natural Phosphodiesterase-4 inhibitors with Potential anti-inflammatory activities from *Millettia dielsiana*

Vu Thi Thu Le[1], Hoang Van Hung[2], Nguyen Xuan Ha[3], Cao Hong Le[1], Pham Thi Hong Minh[3,4] and Do Tien Lam[3,4*]

[1] Thai Nguyen University of Agriculture and Forestry, Thai Nguyen University, Quyet Thang, Thai Nguyen 24119, Viet Nam
[2] Thai Nguyen University-Lao Cai Campus, Thai Nguyen University, Lao Cai City, Lao Cai Province 31000, Vietnam
[3] Institute of Natural Products Chemistry, Vietnam Academy of Science and Technology, 18 Hoang Quoc Viet, Cau Giay, Hanoi 10072, Viet Nam
[4] Faculty of Chemistry, Graduate University of Science and Technology, Vietnam Academy of Science and Technology, 18 Hoang Quoc Viet, Cau Giay, Hanoi 10072, Viet Nam
* Correspondence: dotienlam198@gmail.com; Tel.: (+84)989856515

Table S1: *In silico* Docking of isolated compounds from *Millettia dielsiana* against PDE4A protein

Compounds	Name	ΔG_{dock} (kcal/mol)
D38	Millesianin F	-11.69
D50	5,7,4'-trihydroxyisoflavone 7-O-β-D-apiofuranosyl-(1→6)-β-D-glucopyranoside	-11.68
D45	7-hydroxy-4',8-dimethoxyisoflavone 7-O-β-D-apiofuranosyl-(1→6)-β-D-glucopyranoside	-10.97
D47	Odoratin-7-O-β-D-glucopyranoside	-10.86
D41	Claclrastin-7-O-β-D-glucopyranoside	-10.77
D39	Millesianin G	-10.73
D42	7-hydroxy-4',6 dimethoxyisoflavone-7-O-β-D-apiofuranosyl-(1→6)-β-D-glucopyranoside	-10.57
D23	Hydroxy-6-methoxy-3,4-methylenedioxy-8,3,3-dimethylallyl-isoflavone	-9.97
D19	Ichthynone	-9.93
D46	Glycitin	-9.70
D25	Millesianin D	-9.59
D24	Millesianin H	-9.47
D48	Dalpatin	-9.45
D30	Millesianin C	-9.38
D18	Barbigerone	-9.31
D49	Genistin	-9.28
D40	Daidzin	-9.22
D29	Millesianin B	-9.13
D26	Millesianin I	-9.11
D20	Durmillone	-9.02
D7	Gallocatechin	-8.92
D34	Caviunin	-8.91

D4	Isoliquiritigenin	-8.83
D5	Liquiritigenin	-8.82
D1	Mildiside A	-8.74
D3	Ononin	-8.67
D36	Daidzein	-8.60
D35	Cladrastin	-8.54
D37	Hernancorizin	-8.54
D28	Millesianin A	-8.53
D8	Catechin	-8.52
D10	Tupichinol C	-8.51
D17	Durallone	-8.45
D21	Methoxycalpogonium isoflavone A	-8.44
D11	(+)-Epicatechin	-8.39
D2	Formononetin	-8.39
D32	Millesianin E	-8.39
D22	Calopogonium isoflavone A	-8.33
D44	7-hydroxy-4’,8-dimethoxyisoflavone-7-O-β-D-glucopyranoside	-8.23
D27	Dielsianone	-8.21
D9	(3S)-Vestitol	-8.15
D6	Naringenin	-7.99
D43	Wistin	-7.82
D33	Afromosin	-7.76
D15	*Trans*-3-O-p-hydroxycinnamoyl ursolic acid	-7.06
D14	*Trans*-Ferulic acid	-6.80
D13	Protocatechuic acid	-5.51

Table S2: *In silico* Docking of isolated compounds from *Millettia dielsiana* against PDE4B protein

Compounds	Name	ΔG_{dock} (kcal/mol)
D38	Millesianin F	-11.26
D50	5,7,4'-trihydroxyisoflavone 7-O-β-D-apiofuranosyl-(1→6)-β-D-glucopyranoside	-13.01
D45	7-hydroxy-4',8-dimethoxyisoflavone 7-O-β-D-apiofuranosyl-(1→6)-β-D-glucopyranoside	-10.97
D47	Odoratin-7-O-β-D-glucopyranoside	-10.86
D41	Claclrastin-7-O-β-D-glucopyranoside	-10.77
D39	Millesianin G	-10.73
D42	7-hydroxy-4',6 dimethoxyisoflavone-7-O-β-D-apiofuranosyl-(1→6)-β-D-glucopyranoside	-10.57
D23	Hydroxy-6-methoxy-3,4-methylenedioxy-8,3,3-dimethylallyl-isoflavone	-8.95
D19	Ichthynone	-9.54
D46	Glycitin	-8.34
D25	Millesianin D	-8.75
D24	Millesianin H	-8.54
D48	Dalpatin	-8.83
D30	Millesianin C	-9.27
D18	Barbigerone	-8.6
D49	Genistin	-9.63
D40	Daidzin	-9.6
D29	Millesianin B	-8.65
D26	Millesianin I	-8.96
D20	Durmillone	-8.89
D7	Gallocatechin	-8.55
D34	Caviunin	-7.87
D4	Isoliquiritigenin	-8.52
D5	Liquiritigenin	-8.5
D1	Mildiside A	-7.95
D3	Ononin	-7.8
D36	Daidzein	-8.98
D35	Cladrastin	-8.44
D37	Hernancorizin	-8.29
D28	Millesianin A	-8.25
D8	Catechin	-8.06
D10	Tupichinol C	-8.61
D17	Durallone	-8.1
D21	Methoxycalpogonium isoflavone A	-7.45
D11	(+)-Epicatechin	-8.06
D2	Formononetin	-8.73
D32	Millesianin E	-9.72
D22	Calopogonium isoflavone A	-8.73
D44	7-hydroxy-4',8-dimethoxyisoflavone-7-O-β-D-glucopyranoside	-8.78
D27	Dielsianone	-8.01

D9	(3S)-Vestitol	-8.15
D6	Naringenin	-7.59
D43	Wistin	-8.53
D33	Afromosin	-8.64
D15	*Trans*-3-O-p-hydroxycinnamoyl ursolic acid	-5.27
D14	*Trans*-Ferulic acid	-6.93
D13	Protocatechuic acid	-5.46

Table S3: *In silico* Docking of isolated compoundsfrom *Millettia dielsiana* against PDE4D protein

Compounds	Name	ΔGdock (kcal/mol)
D38	Millesianin F	-11.4
D50	5,7,4'-trihydroxyisoflavone 7-O-β-D-apiofuranosyl-(1→6)-β-D-glucopyranoside	-7.12
D45	7-hydroxy-4',8-dimethoxyisoflavone 7-O-β-D-apiofuranosyl-(1→6)-β-D-glucopyranoside	-9.99
D47	Odoratin-7-O-β-D-glucopyranoside	-9.53
D41	Claclrastin-7-O-β-D-glucopyranoside	-10.78
D39	Millesianin G	-12.12
D42	7-hydroxy-4',6 dimethoxyisoflavone-7-O-β-D-apiofuranosyl-(1→6)-β-D-glucopyranoside	-11.08
D23	Hydroxy-6-methoxy-3,4-methylenedioxy-8,3,3-dimethylallyl-isoflavone	-9.61
D19	Ichthynone	-9.63
D46	Glycitin	-10.53
D25	Millesianin D	-9.54
D24	Millesianin H	-9.16
D48	Dalpatin	-9.04
D30	Millesianin C	-9.14
D18	Barbigerone	-8.83
D49	Genistin	-10.21
D40	Daidzin	-10.06
D29	Millesianin B	-8.85
D26	Millesianin I	-8.69
D20	Durmillone	-8.63
D7	Gallocatechin	-9.04
D34	Caviunin	-8.58
D4	Isoliquiritigenin	-6.95

D5	Liquiritigenin	-8.41
D1	Mildiside A	-8.64
D3	Ononin	-8.52
D36	Daidzein	-8.62
D35	Cladrastin	-9.14
D37	Hernancorizin	-8.47
D28	Millesianin A	-9.19
D8	Catechin	-8.19
D10	Tupichinol C	-8.79
D17	Durallone	-8.16
D21	Methoxycalpogonium isoflavone A	-8.19
D11	(+)-Epicatechin	-8.03
D2	Formononetin	-8.36
D32	Millesianin E	-9.02
D22	Calopogonium isoflavone A	-8.06
D44	7-hydroxy-4’,8-dimethoxyisoflavone-7-O-β-D-glucopyranoside	-9.67
D27	Dielsianone	-8.02
D9	(3S)-Vestitol	-8.27
D6	Naringenin	-7.28
D43	Wistin	-9.19
D33	Afromosin	-8.61
D15	Trans-3-O-p-hydroxycinnamoyl ursolic acid	-7.6
D14	Trans-Ferulic acid	-7.45
D13	Protocatechuic acid	-5.8

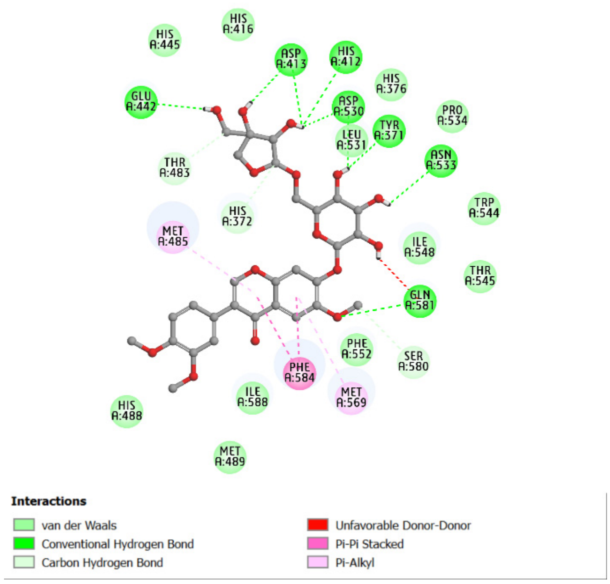

D38

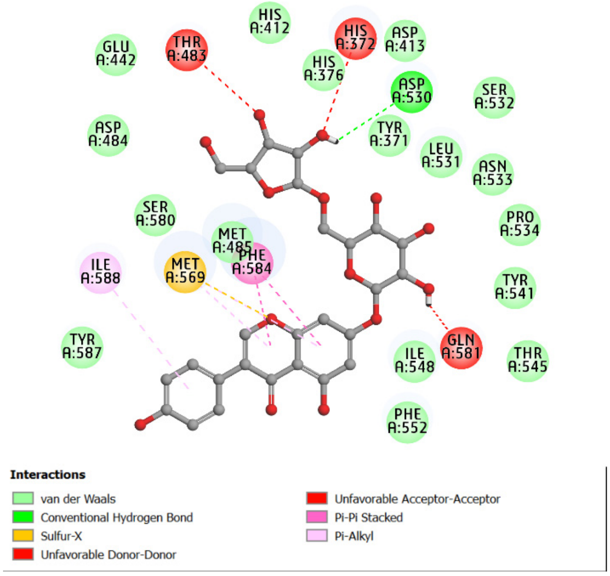

D50

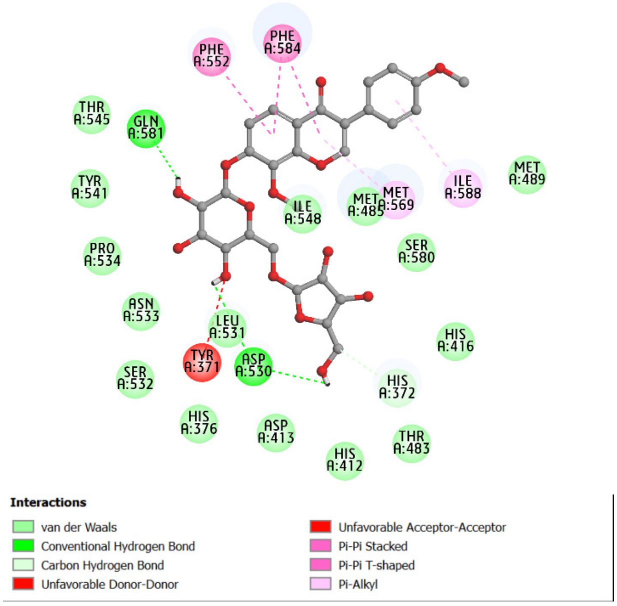

D45

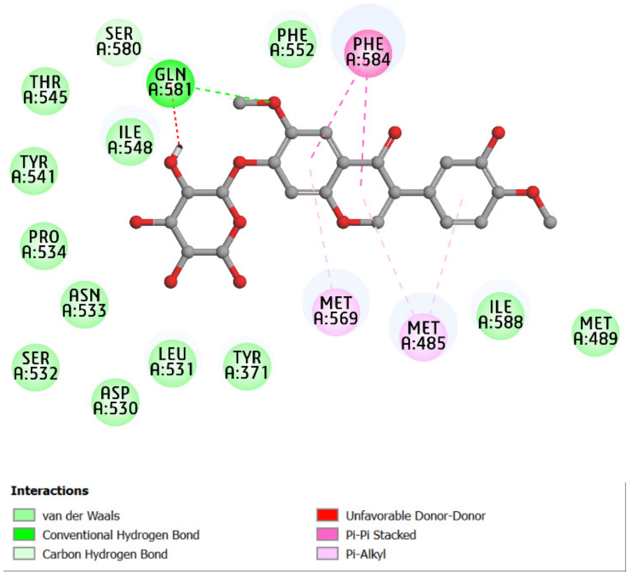

D47

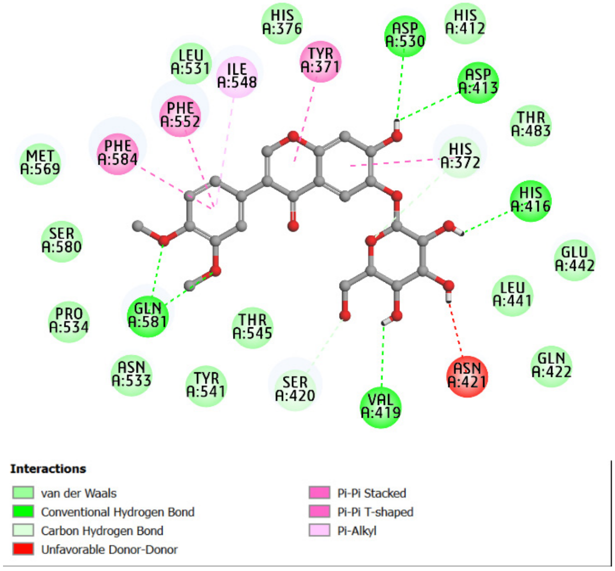

D41

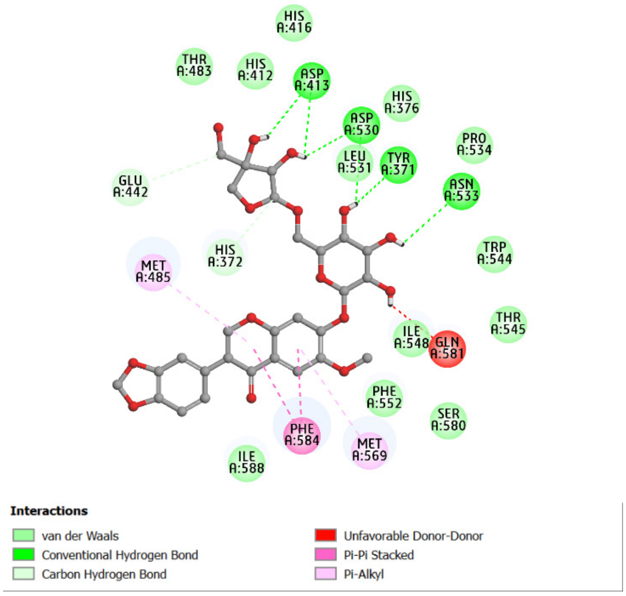

D39

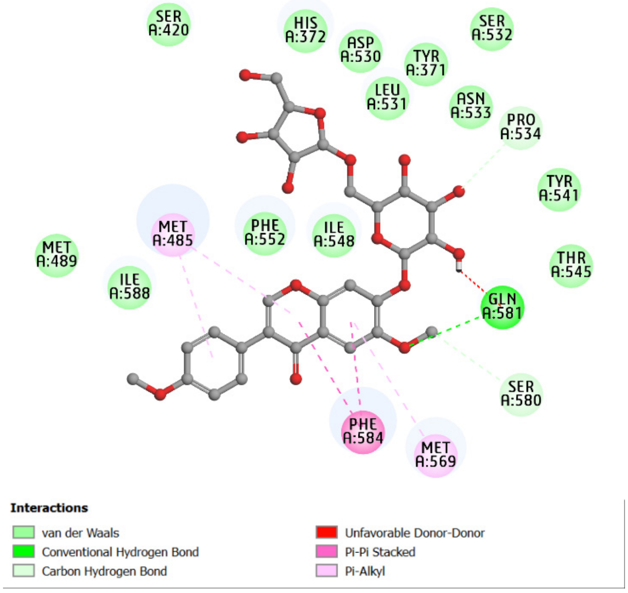

D42

Figure S1. 2D representation of binding interaction of the hit compounds and the amino acid residues of PDE4A enzyme (PDB ID: 3TVX).

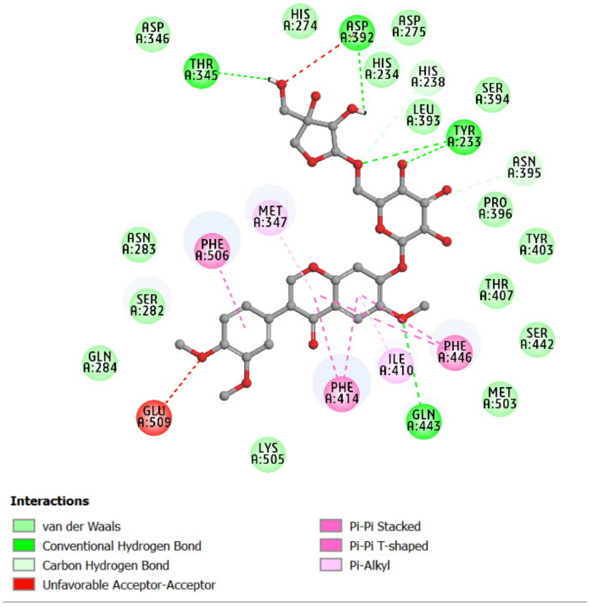

D38

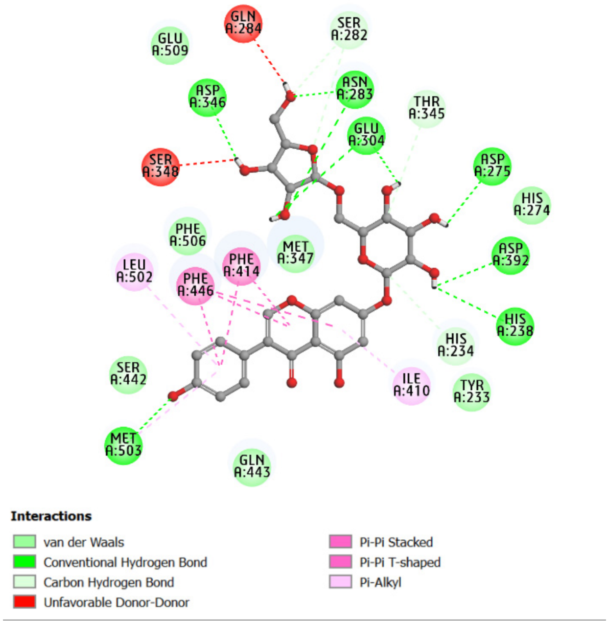

D50

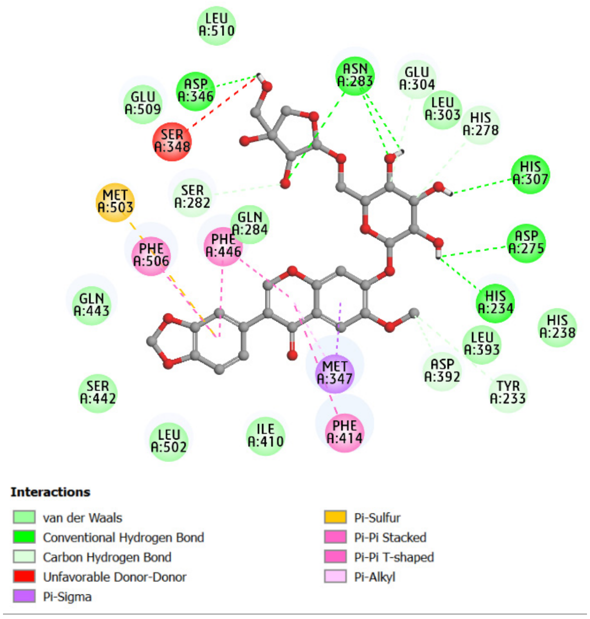

D39

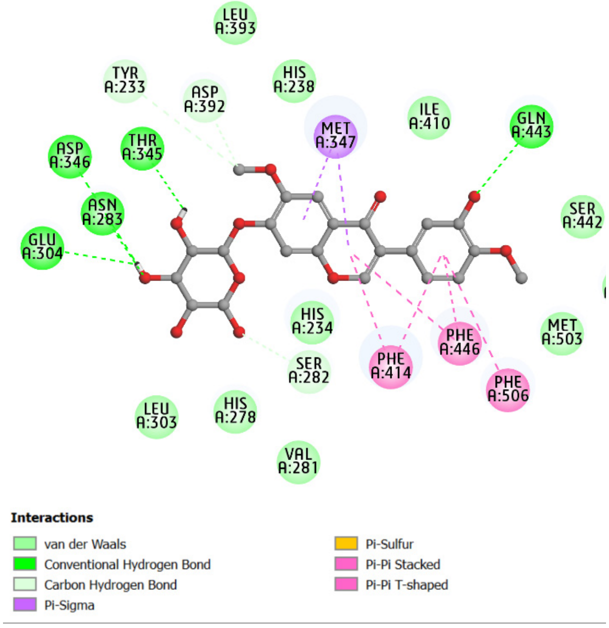

D47

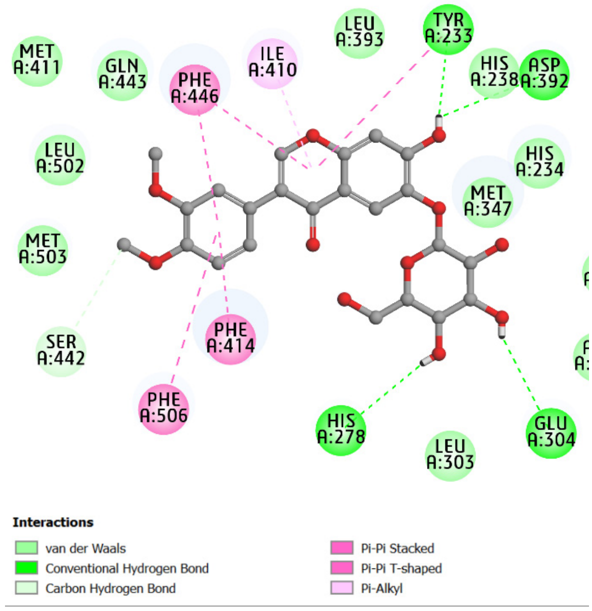

D41

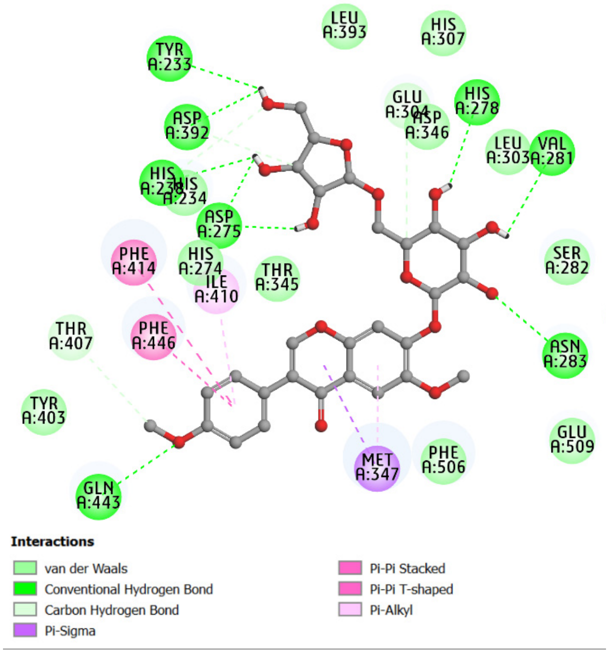

D42

Figure S2. 2D representation of binding interaction of the hit compounds and the amino acid residues of PDE4B enzyme (PDB ID: 3W5E).

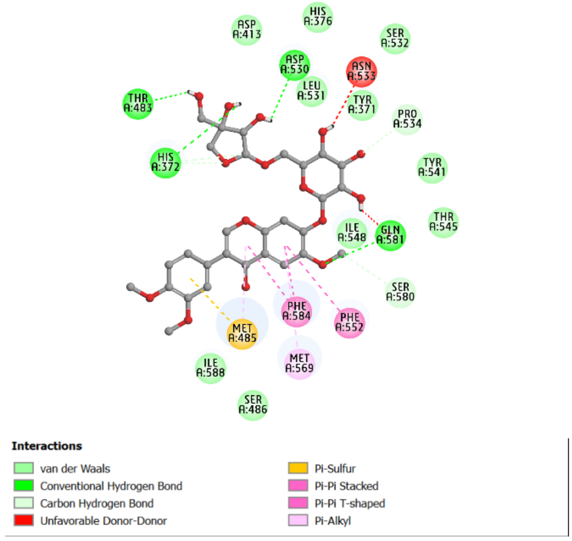

D38

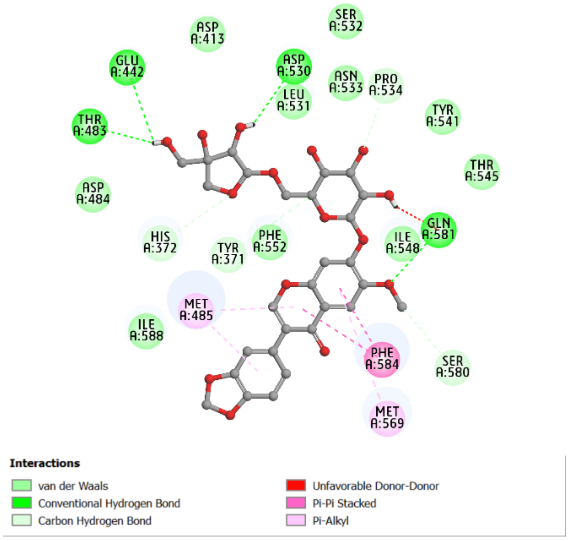

D39

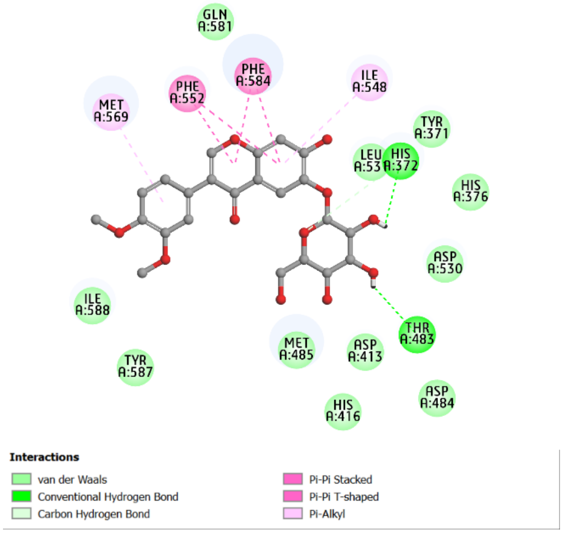

D41

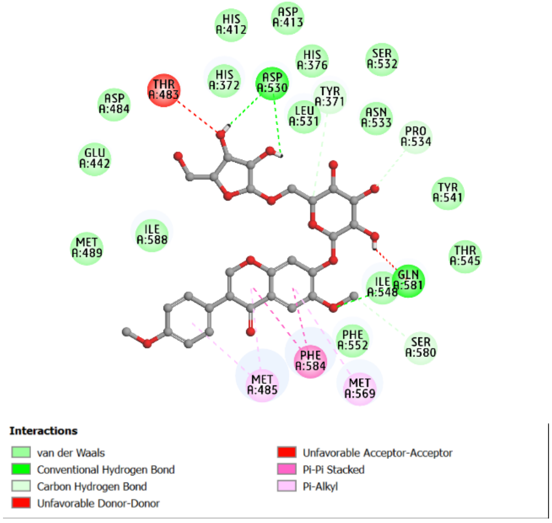

D42

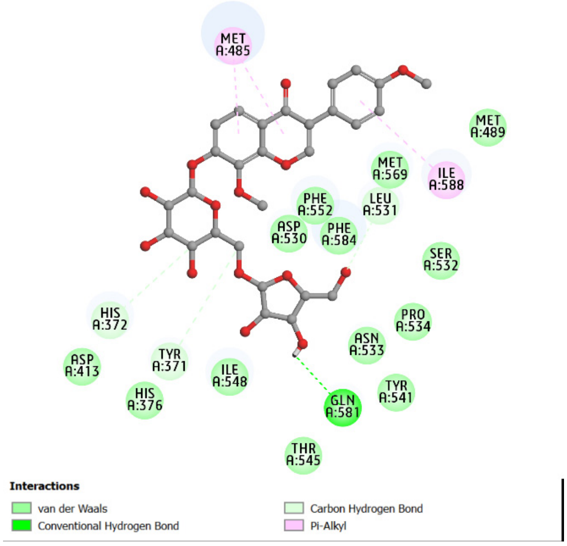

D45

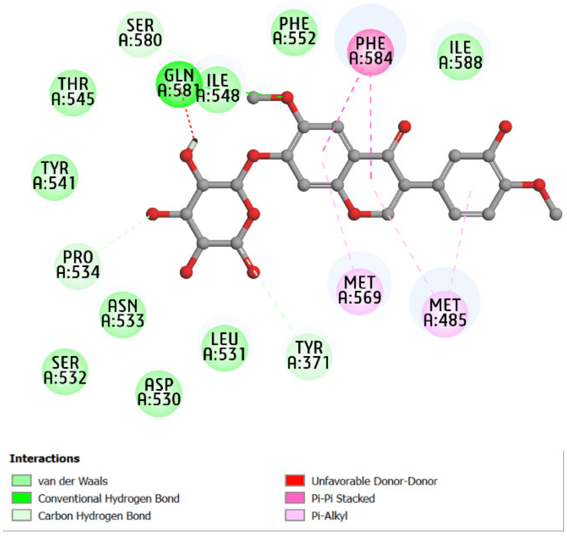

D47

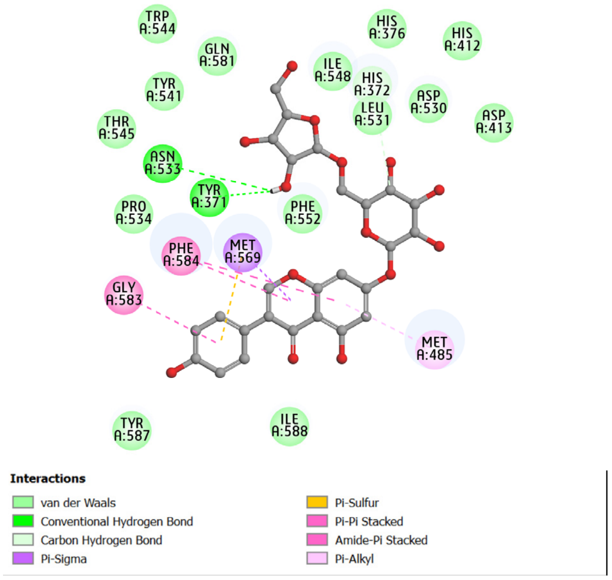

D50

Figure S3. 2D interaction diagrams of the studied compounds from MD-refined structures of these complexes with PDE4A were analyzed at a time of 100 ns

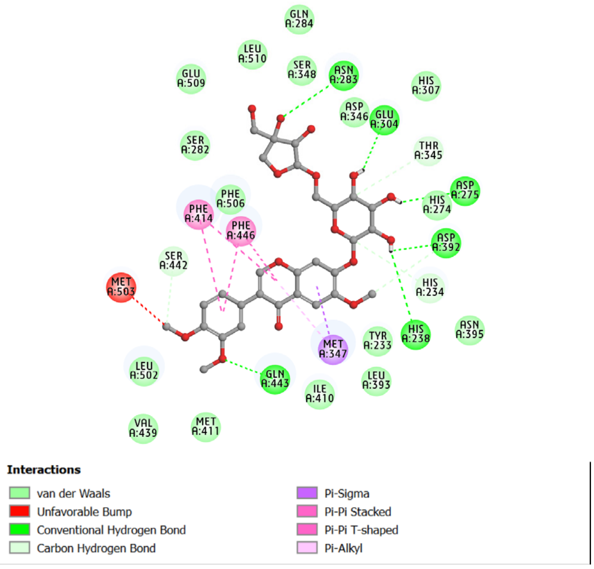

D38

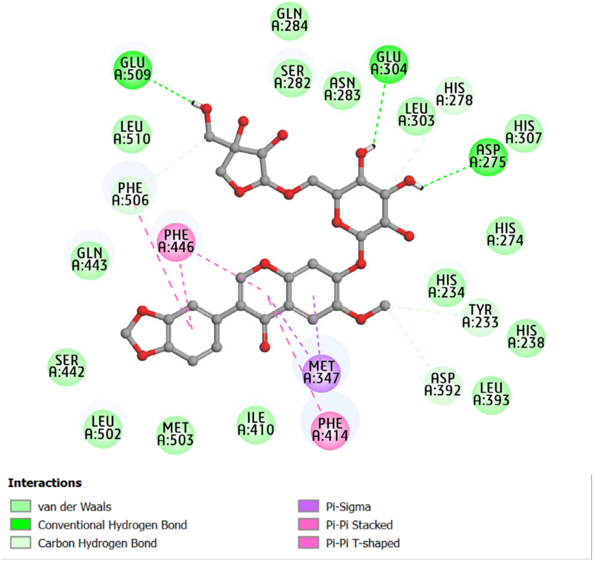

D39

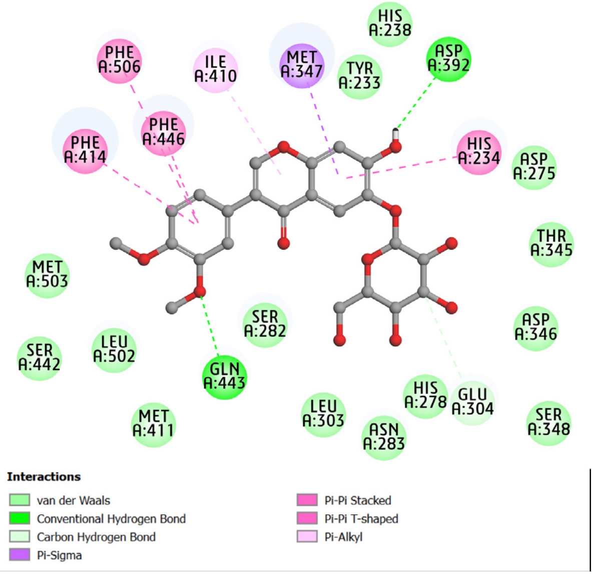

D41

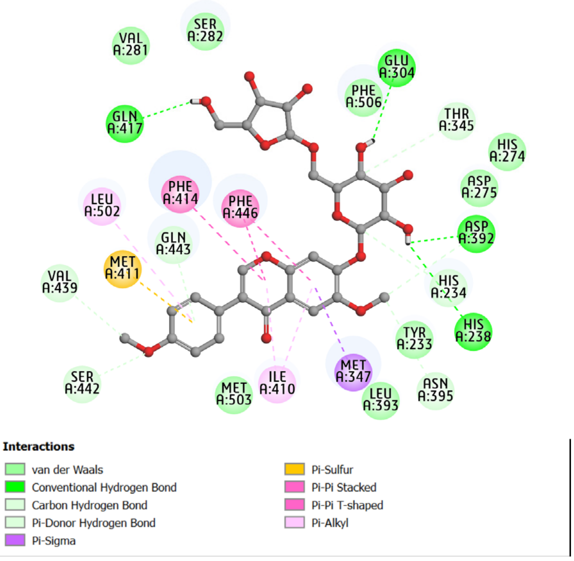

D42

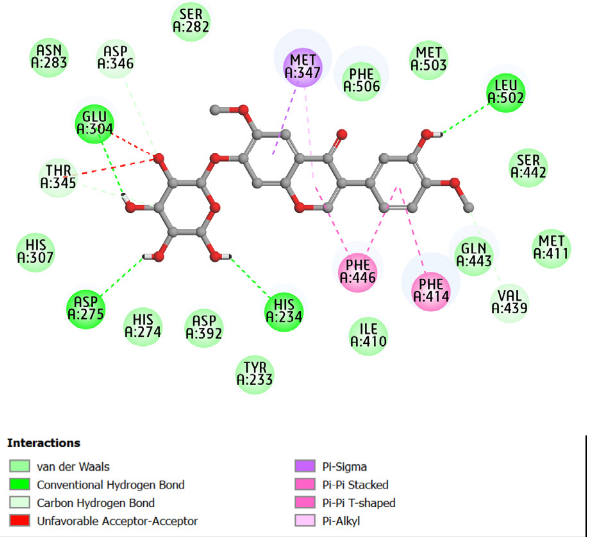

D47

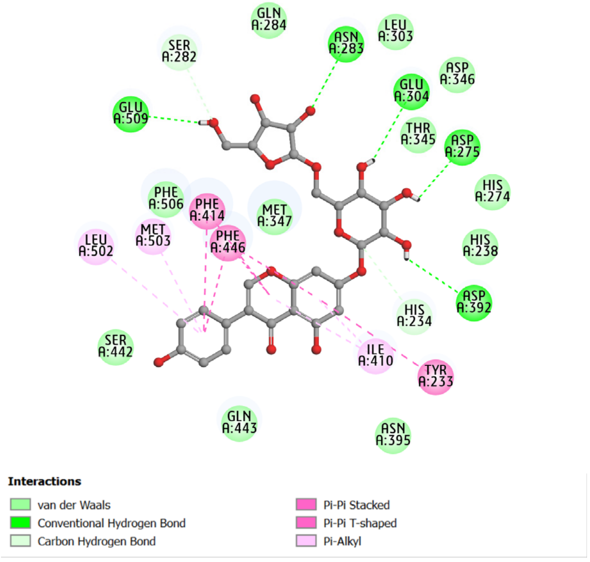

D50

Figure S4. 2D interaction diagrams of the studied compounds from MD-refined structures of these complexes with PDE4B were analyzed at a time of 100 ns

Table S4. Acute oral toxicity prediction obtained by using the ProTox-II web server

**Entry**	**Compound name**	**LD_{50} (mg/kg)**	**Toxicity class**
D38	Millesianin F	5000	5
D39	Millesianin G	5000	5
D41	Claclrastin-7-O-β-D-glucopyranoside	5000	5
D42	7-hydroxy-4’,6 dimethoxyisoflavone-7-O-*β*-D-apiofuranosyl-(1→6)-*β*-D-glucopyranoside	5000	5
D45	7-hydroxy-4’,8-dimethoxyisoflavone 7-O-*β*-D-apiofuranosyl-(1→6)-*β*-D-glucopyranoside	5000	5
D47	Odoratin-7-O-*β*-D-glucopyranoside	5000	5
D50	5,7,4’-trihydroxyisoflavone 7-*O*-*β*-D-apiofuranosyl-(1→6)-*β*-D-glucopyranoside	5000	5
Ref	3KQ	2000	4
	NVW	780	4
	pentoxifylline	780	4

Table S5. Organ toxicity, toxicological endpoints, and toxicological pathways predicted activity calculated using the ProTox-II web server

D39

Toxicity Model Report

Classification	Target	Shorthand	Prediction	Probability
Organ toxicity	Hepatotoxicity	dili	Inactive	0.88
Toxicity end points	Carcinogenicity	carcino	Inactive	0.78
Toxicity end points	Immunotoxicity	immuno	Active	0.99
Toxicity end points	Mutagenicity	mutagen	Inactive	0.57
Toxicity end points	Cytotoxicity	cyto	Inactive	0.75
Tox21-Nuclear receptor signalling pathways	Aryl hydrocarbon Receptor (AhR)	nr_ahr	Inactive	0.78
Tox21-Nuclear receptor signalling pathways	Androgen Receptor (AR)	nr_ar	Inactive	0.98
Tox21-Nuclear receptor signalling pathways	Androgen Receptor Ligand Binding Domain (AR-LBD)	nr_ar_lbd	Inactive	0.94
Tox21-Nuclear receptor signalling pathways	Aromatase	nr_aromatase	Inactive	0.82
Tox21-Nuclear receptor signalling pathways	Estrogen Receptor Alpha (ER)	nr_er	Inactive	0.85
Tox21-Nuclear receptor signalling pathways	Estrogen Receptor Ligand Binding Domain (ER-LBD)	nr_er_lbd	Inactive	0.97
Tox21-Nuclear receptor signalling pathways	Peroxisome Proliferator Activated Receptor Gamma (PPAR-Gamma)	nr_ppar_gamma	Inactive	0.87
Tox21-Stress response pathways	Nuclear factor (erythroid-derived 2)-like 2/antioxidant responsive element (nrf2/ARE)	sr_are	Inactive	0.85
Tox21-Stress response pathways	Heat shock factor response element (HSE)	sr_hse	Inactive	0.85
Tox21-Stress response pathways	Mitochondrial Membrane Potential (MMP)	sr_mmp	Inactive	0.72
Tox21-Stress response pathways	Phosphoprotein (Tumor Supressor) p53	sr_p53	Inactive	0.50
Tox21-Stress response pathways	ATPase family AAA domain-containing protein 5 (ATAD5)	sr_atad5	Inactive	0.90

D38

Toxicity Model Report

Classification	Target	Shorthand	Prediction	Probability
Organ toxicity	Hepatotoxicity	dili	Inactive	0.88
Toxicity end points	Carcinogenicity	carcino	Inactive	0.83
Toxicity end points	Immunotoxicity	immuno	Active	0.99
Toxicity end points	Mutagenicity	mutagen	Inactive	0.67
Toxicity end points	Cytotoxicity	cyto	Inactive	0.67
Tox21-Nuclear receptor signalling pathways	Aryl hydrocarbon Receptor (AhR)	nr_ahr	Inactive	0.82
Tox21-Nuclear receptor signalling pathways	Androgen Receptor (AR)	nr_ar	Inactive	0.98
Tox21-Nuclear receptor signalling pathways	Androgen Receptor Ligand Binding Domain (AR-LBD)	nr_ar_lbd	Inactive	0.93
Tox21-Nuclear receptor signalling pathways	Aromatase	nr_aromatase	Inactive	0.76
Tox21-Nuclear receptor signalling pathways	Estrogen Receptor Alpha (ER)	nr_er	Inactive	0.85
Tox21-Nuclear receptor signalling pathways	Estrogen Receptor Ligand Binding Domain (ER-LBD)	nr_er_lbd	Inactive	0.92
Tox21-Nuclear receptor signalling pathways	Peroxisome Proliferator Activated Receptor Gamma (PPAR-Gamma)	nr_ppar_gamma	Inactive	0.82
Tox21-Stress response pathways	Nuclear factor (erythroid-derived 2)-like 2/antioxidant responsive element (nrf2/ARE)	sr_are	Inactive	0.86
Tox21-Stress response pathways	Heat shock factor response element (HSE)	sr_hse	Inactive	0.86
Tox21-Stress response pathways	Mitochondrial Membrane Potential (MMP)	sr_mmp	Inactive	0.69
Tox21-Stress response pathways	Phosphoprotein (Tumor Supressor) p53	sr_p53	Active	0.54
Tox21-Stress response pathways	ATPase family AAA domain-containing protein 5 (ATAD5)	sr_atad5	Inactive	0.88

D41

Toxicity Model Report

Classification	Target	Shorthand	Prediction	Probability
Organ toxicity	Hepatotoxicity	dili	Inactive	0.83
Toxicity end points	Carcinogenicity	carcino	Inactive	0.93
Toxicity end points	Immunotoxicity	immuno	Active	0.96
Toxicity end points	Mutagenicity	mutagen	Inactive	0.77
Toxicity end points	Cytotoxicity	cyto	Active	0.50
Tox21-Nuclear receptor signalling pathways	Aryl hydrocarbon Receptor (AhR)	nr_ahr	Active	0.61
Tox21-Nuclear receptor signalling pathways	Androgen Receptor (AR)	nr_ar	Inactive	0.99
Tox21-Nuclear receptor signalling pathways	Androgen Receptor Ligand Binding Domain (AR-LBD)	nr_ar_lbd	Inactive	0.99
Tox21-Nuclear receptor signalling pathways	Aromatase	nr_aromatase	Inactive	0.99
Tox21-Nuclear receptor signalling pathways	Estrogen Receptor Alpha (ER)	nr_er	Inactive	0.98
Tox21-Nuclear receptor signalling pathways	Estrogen Receptor Ligand Binding Domain (ER-LBD)	nr_er_lbd	Inactive	0.99
Tox21-Nuclear receptor signalling pathways	Peroxisome Proliferator Activated Receptor Gamma (PPAR-Gamma)	nr_ppar_gamma	Inactive	0.99
Tox21-Stress response pathways	Nuclear factor (erythroid-derived 2)-like 2/antioxidant responsive element (nrf2/ARE)	sr_are	Inactive	0.99
Tox21-Stress response pathways	Heat shock factor response element (HSE)	sr_hse	Inactive	0.99
Tox21-Stress response pathways	Mitochondrial Membrane Potential (MMP)	sr_mmp	Inactive	0.99
Tox21-Stress response pathways	Phosphoprotein (Tumor Supressor) p53	sr_p53	Inactive	0.90
Tox21-Stress response pathways	ATPase family AAA domain-containing protein 5 (ATAD5)	sr_atad5	Inactive	0.99

D42

Toxicity Model Report

Classification	Target	Shorthand	Prediction	Probability
Organ toxicity	Hepatotoxicity	dili	Inactive	0.84
Toxicity end points	Carcinogenicity	carcino	Inactive	0.85
Toxicity end points	Immunotoxicity	immuno	Active	0.97
Toxicity end points	Mutagenicity	mutagen	Inactive	0.73
Toxicity end points	Cytotoxicity	cyto	Inactive	0.64
Tox21-Nuclear receptor signalling pathways	Aryl hydrocarbon Receptor (AhR)	nr_ahr	Inactive	0.57
Tox21-Nuclear receptor signalling pathways	Androgen Receptor (AR)	nr_ar	Inactive	0.97
Tox21-Nuclear receptor signalling pathways	Androgen Receptor Ligand Binding Domain (AR-LBD)	nr_ar_lbd	Inactive	0.99
Tox21-Nuclear receptor signalling pathways	Aromatase	nr_aromatase	Inactive	0.97
Tox21-Nuclear receptor signalling pathways	Estrogen Receptor Alpha (ER)	nr_er	Inactive	0.91
Tox21-Nuclear receptor signalling pathways	Estrogen Receptor Ligand Binding Domain (ER-LBD)	nr_er_lbd	Inactive	0.99
Tox21-Nuclear receptor signalling pathways	Peroxisome Proliferator Activated Receptor Gamma (PPAR-Gamma)	nr_ppar_gamma	Inactive	0.93
Tox21-Stress response pathways	Nuclear factor (erythroid-derived 2)-like 2/antioxidant responsive element (nrf2/ARE)	sr_are	Inactive	0.85
Tox21-Stress response pathways	Heat shock factor response element (HSE)	sr_hse	Inactive	0.85
Tox21-Stress response pathways	Mitochondrial Membrane Potential (MMP)	sr_mmp	Inactive	0.85
Tox21-Stress response pathways	Phosphoprotein (Tumor Supressor) p53	sr_p53	Inactive	0.55
Tox21-Stress response pathways	ATPase family AAA domain-containing protein 5 (ATAD5)	sr_atad5	Inactive	0.92

D45

Toxicity Model Report

Classification	Target	Shorthand	Prediction	Probability
Organ toxicity	Hepatotoxicity	dili	Inactive	0.84
Toxicity end points	Carcinogenicity	carcino	Inactive	0.85
Toxicity end points	Immunotoxicity	immuno	Active	0.87
Toxicity end points	Mutagenicity	mutagen	Inactive	0.73
Toxicity end points	Cytotoxicity	cyto	Inactive	0.64
Tox21-Nuclear receptor signalling pathways	Aryl hydrocarbon Receptor (AhR)	nr_ahr	Inactive	0.57
Tox21-Nuclear receptor signalling pathways	Androgen Receptor (AR)	nr_ar	Inactive	0.97
Tox21-Nuclear receptor signalling pathways	Androgen Receptor Ligand Binding Domain (AR-LBD)	nr_ar_lbd	Inactive	0.99
Tox21-Nuclear receptor signalling pathways	Aromatase	nr_aromatase	Inactive	0.97
Tox21-Nuclear receptor signalling pathways	Estrogen Receptor Alpha (ER)	nr_er	Inactive	0.91
Tox21-Nuclear receptor signalling pathways	Estrogen Receptor Ligand Binding Domain (ER-LBD)	nr_er_lbd	Inactive	0.99
Tox21-Nuclear receptor signalling pathways	Peroxisome Proliferator Activated Receptor Gamma (PPAR-Gamma)	nr_ppar_gamma	Inactive	0.93
Tox21-Stress response pathways	Nuclear factor (erythroid-derived 2)-like 2/antioxidant responsive element (nrf2/ARE)	sr_are	Inactive	0.85
Tox21-Stress response pathways	Heat shock factor response element (HSE)	sr_hse	Inactive	0.85
Tox21-Stress response pathways	Mitochondrial Membrane Potential (MMP)	sr_mmp	Inactive	0.85
Tox21-Stress response pathways	Phosphoprotein (Tumor Supressor) p53	sr_p53	Inactive	0.55
Tox21-Stress response pathways	ATPase family AAA domain-containing protein 5 (ATAD5)	sr_atad5	Inactive	0.92

D47

Toxicity Model Report

Classification	Target	Shorthand	Prediction	Probability
Organ toxicity	Hepatotoxicity	dili	Inactive	0.76
Toxicity end points	Carcinogenicity	carcino	Inactive	0.53
Toxicity end points	Immunotoxicity	immuno	Active	0.98
Toxicity end points	Mutagenicity	mutagen	Inactive	0.69
Toxicity end points	Cytotoxicity	cyto	Inactive	0.91
Tox21-Nuclear receptor signalling pathways	Aryl hydrocarbon Receptor (AhR)	nr_ahr	Active	0.61
Tox21-Nuclear receptor signalling pathways	Androgen Receptor (AR)	nr_ar	Inactive	0.99
Tox21-Nuclear receptor signalling pathways	Androgen Receptor Ligand Binding Domain (AR-LBD)	nr_ar_lbd	Inactive	0.95
Tox21-Nuclear receptor signalling pathways	Aromatase	nr_aromatase	Inactive	0.93
Tox21-Nuclear receptor signalling pathways	Estrogen Receptor Alpha (ER)	nr_er	Inactive	0.71
Tox21-Nuclear receptor signalling pathways	Estrogen Receptor Ligand Binding Domain (ER-LBD)	nr_er_lbd	Inactive	0.77
Tox21-Nuclear receptor signalling pathways	Peroxisome Proliferator Activated Receptor Gamma (PPAR-Gamma)	nr_ppar_gamma	Inactive	0.95
Tox21-Stress response pathways	Nuclear factor (erythroid-derived 2)-like 2/antioxidant responsive element (nrf2/ARE)	sr_are	Inactive	0.88
Tox21-Stress response pathways	Heat shock factor response element (HSE)	sr_hse	Inactive	0.88
Tox21-Stress response pathways	Mitochondrial Membrane Potential (MMP)	sr_mmp	Inactive	0.50
Tox21-Stress response pathways	Phosphoprotein (Tumor Supressor) p53	sr_p53	Inactive	0.80
Tox21-Stress response pathways	ATPase family AAA domain-containing protein 5 (ATAD5)	sr_atad5	Inactive	0.66

D50

Toxicity Model Report

Classification	Target	Shorthand	Prediction	Probability
Organ toxicity	Hepatotoxicity	dili	Inactive	0.82
Toxicity end points	Carcinogenicity	carcino	Inactive	0.79
Toxicity end points	Immunotoxicity	immuno	Inactive	0.54
Toxicity end points	Mutagenicity	mutagen	Inactive	0.72
Toxicity end points	Cytotoxicity	cyto	Inactive	0.72
Tox21-Nuclear receptor signalling pathways	Aryl hydrocarbon Receptor (AhR)	nr_ahr	Inactive	0.87
Tox21-Nuclear receptor signalling pathways	Androgen Receptor (AR)	nr_ar	Inactive	0.86
Tox21-Nuclear receptor signalling pathways	Androgen Receptor Ligand Binding Domain (AR-LBD)	nr_ar_lbd	Inactive	0.98
Tox21-Nuclear receptor signalling pathways	Aromatase	nr_aromatase	Inactive	0.98
Tox21-Nuclear receptor signalling pathways	Estrogen Receptor Alpha (ER)	nr_er	Inactive	0.79
Tox21-Nuclear receptor signalling pathways	Estrogen Receptor Ligand Binding Domain (ER-LBD)	nr_er_lbd	Inactive	0.99
Tox21-Nuclear receptor signalling pathways	Peroxisome Proliferator Activated Receptor Gamma (PPAR-Gamma)	nr_ppar_gamma	Inactive	0.93
Tox21-Stress response pathways	Nuclear factor (erythroid-derived 2)-like 2/antioxidant responsive element (nrf2/ARE)	sr_are	Inactive	0.89
Tox21-Stress response pathways	Heat shock factor response element (HSE)	sr_hse	Inactive	0.89
Tox21-Stress response pathways	Mitochondrial Membrane Potential (MMP)	sr_mmp	Inactive	0.88
Tox21-Stress response pathways	Phosphoprotein (Tumor Supressor) p53	sr_p53	Inactive	0.57
Tox21-Stress response pathways	ATPase family AAA domain-containing protein 5 (ATAD5)	sr_atad5	Inactive	0.95
